# Esophageal ILC2s mediate abnormal epithelial remodeling in eosinophilic esophagitis via Areg-EGFR signaling

**DOI:** 10.1038/s41423-024-01242-x

**Published:** 2024-12-09

**Authors:** MinYeong Lim, Taesoo Kim, Hyesung Kim, Bo Gun Jang, Jae Kyung Myung, Hye Young Kim

**Affiliations:** 1 Laboratory of Mucosal Immunology, Department of Biomedical Sciences, Seoul National University College of Medicine, Seoul, South Korea; 2 Institute of Allergy and Clinical Immunology, Seoul National University Medical Research Center, Seoul, South Korea; 3 Department of Biological Sciences, SRC Center for Immune Research on Nonlymphoid Organs, Sungkyunkwan University, Suwon, South Korea; 4 Department of Life Science and Multitasking Macrophage Research Center, Ewha Womans University, Seoul, South Korea; 5 Jeju National University College of Medicine, Jeju, South Korea; 6 Department of Pathology, Jeju National University College of Medicine and Jeju National University Hospital, Jeju, South Korea; 7 Department of Pathology, Hanyang University College of Medicine, Seoul, South Korea

**Keywords:** Eosinophilic esophagitis (EoE), Innate lymphoid cells (ILCs), Amphiregulin (Areg), Epidermal growth factor receptor (EGFR), Epidermal hyperplasia, Allergic disorder, Innate lymphoid cells, Mucosal immunology, Allergy

## Abstract

Eosinophilic esophagitis (EoE) is a chronic allergic disorder characterized by eosinophilia and epithelial thickening, resulting in dysphagia. While emerging evidence implicates increased frequencies of group 2 innate lymphoid cells (ILC2s) and increased interleukin (IL)-33 expression in EoE pathogenesis, the precise mechanisms remain unclear. In this study, we investigated the role of ILC2s in EoE pathogenesis. We observed an abundance of KLRG1^+^ ILC2s in the esophagi of healthy mice, with their numbers significantly increasing in murine EoE models and humans. Using a murine EoE model, we demonstrated the recapitulation of EoE-associated features, including basal-cell hyperproliferation, epithelial thickening, and eosinophilia. Notably, these characteristics are absent in ILC-deficient mice, whereas mice lacking IL-5 or eosinophils display epithelial defects, highlighting the pivotal role of ILC2s in EoE pathogenesis. Further investigations revealed increased amphiregulin (Areg) production by esophageal ILC2s in mice. The administration of Areg induced epithelial defects similar to those observed in EoE. Mechanistic studies using human esophageal cell lines revealed Areg-induced phosphorylation of epidermal growth factor receptor (EGFR). Significatntly, treatment with anti-Areg agents and EGFR inhibitors effectively attenuated EoE development, highlighting the therapeutic potential of targeting the Areg-EGFR axis.

## Introduction

Eosinophilic esophagitis (EoE) has emerged as a chronic allergic disorder that has significantly increased in prevalence over the past two decades and is now recognized as a prominent form of esophageal dysfunction [1]. Characterized by persistent eosinophilia and type-2 inflammatory responses within the mucosa, EoE manifests through distinct pathological features, including basal-cell hyperproliferation, epithelial thickening, and muscular hypertrophy [2]. These aberrations contribute to the development of strictures and rings in the esophageal mucosa [3, 4]. Basal cells, which are located in the deepest layer of the esophageal epithelium, undergo asymmetric division and differentiation to uphold the mucosal lining, which is critical for esophageal function and homeostasis [5, 6]. However, disruptions in basal cell proliferation and differentiation, prompted by factors such as food intake, infection, and inflammation, can precipitate pathological conditions such as EoE [7].

Recent investigations have shed light on the role of innate lymphoid cells (ILCs), particularly group-2 ILCs (ILC2s), as pivotal contributors to type 2 inflammation, which has historically been attributed to adaptive T helper-2 (Th2) cells [8]. In many mucosal tissues, ILC2s respond to environmental cues by secreting type 2 cytokines, including interleukins (IL)-4, IL-5, IL-13, and amphiregulin (Areg), upon activation by alarmins such as IL-25, thymic stromal lymphopoietin (TSLP), and IL-33 [8–10]. Dysregulation of ILC2s has been implicated in severe type 2 inflammatory conditions, including EoE, as evidenced by the elevated ILC2 frequency in active EoE patients compared with healthy individuals and those in remission [11, 12]. Aregs, produced by ILC2s, interact with epidermal growth factor receptor (EGFR) [13], suggesting a potential role in regulating epithelial cell proliferation and differentiation. This hypothesis is supported by studies demonstrating the involvement of ILC2-derived Aregs in maintaining epithelial integrity in other mucosal tissues [14, 15].

However, the precise mechanisms underlying the crosstalk between esophageal epithelial cells and ILC2s, particularly in the context of EoE, remain inadequately understood. Our study aims to bridge this knowledge gap by characterizing esophageal ILC2s in both human EoE biopsies and murine models of IL-33-induced EoE. Furthermore, our experiments demonstrated that activated ILC2s directly contribute to abnormal epithelial remodeling in EoE through the Areg-EGFR signaling cascade. This investigation highlights the intricate interplay between ILC2s and esophageal epithelial cells in EoE pathogenesis, offering insights into potential therapeutic targets for further exploration.

## Results

### Characterization of innate lymphoid cells in murine and human esophagus

ILC2s are found in various mucosal tissues, including the lung and gastrointestinal tract. Despite being a small population in the lungs, ILC2s are important for defending against infections and respiratory disorders such as allergic asthma [16, 17]. However, our understanding of the role of esophageal ILCs in health and disease remains limited.

**Fig. 1:**
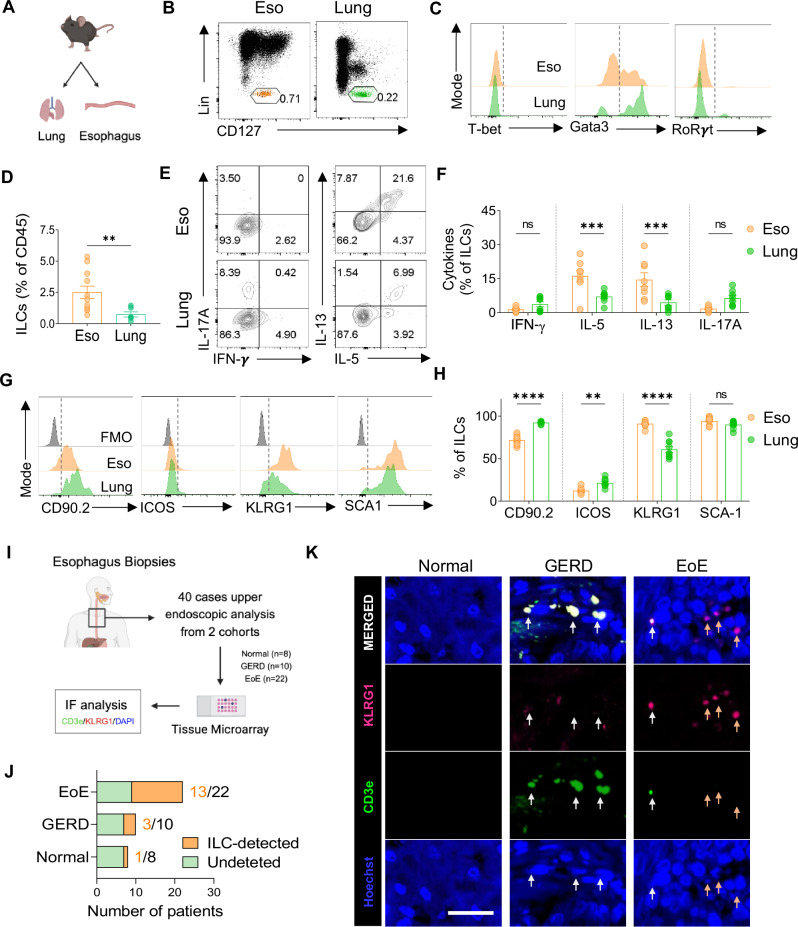
Characterization of ILCs in the mouse esophagus and human EoE patients. **A** Schematic representation of mouse esophageal and lung immune cells analyzed via flow cytometry. **B** FACS plots showing ILCs in esophageal (orange) and lung (green) tissues. **C** Comparison of transcription factor expression in total esophageal and lung ILCs. **D** Frequencies of ILCs in the esophagus and lung. FACS plots (**E**) and frequencies (**F**) of cytokine-producing ILCs in the esophagus and lung. **G** Comparison of surface molecules in total esophageal and lung CD25^+ ^ST2^+^ ILC2s (gray: fluorescence minus one control (FMO)). **H** Frequencies of CD90.2, ICOS, KLRG1, and SCA1^+^ ILC2s in total esophageal and lung CD25^+^ ST2^+^ ILC2s. **I** Study design for human esophageal biopsies analyzed via immunofluorescence. **J** Number of ILCs detected in patients with esophageal diseases. **K** Representative immunofluorescence images from healthy controls and GERD and EoE patients (KLRG1: magenta; CD3e: green; and DAPI: blue). The white arrows indicate CD3^+^ T cells, whereas the orange arrows indicate KLRG1^+^ ILC2s. Scale bars = 50 µm. The data were pooled from at least 2‒3 independent experiments and are presented as the means ± SEMs. *P ≤ 0.05, **P ≤ 0.01, ***P ≤ 0.001, ****P ≤ 0.0001, ns, not significant

We conducted flow cytometry analysis to characterize resident ILCs in the esophagus of healthy mice and compared them with lung-resident ILCs (Fig. 1A–D and Supplementary Fig. 1A-B). Approximately 2% of immune cells in the esophagus were ILCs, whereas less than 1% were ILCs in the lung (Fig. 1D). Conversely, CD4^+^ T cells were frequent in the lung but rare in the esophagus (Supplementary Fig. 1C). Notably, GATA3^+^ ILC2s predominated in both tissues, with higher abundance in the esophagus (Fig. 1C). Compared with lung ILCs, esophageal ILCs tended to produce the ILC2-associated cytokines IL-5 and IL-13 (Fig. 1E-F). Further characterization of esophageal ILC2s revealed distinct surface marker expression patterns, including high KLRG1 expression compared with that of lung ILC2s, indicating potential functional differences (Fig. 1G-H and Supplementary Fig. 1D).

To assess the presence of ILCs in the esophagus of healthy patients, we conducted immunofluorescence analysis on endoscopic biopsy samples from 8 healthy patients for CD3e^−^KLRG1^+^ILC2s. KLRG1 was chosen as a comarker because of its high expression in normal esophageal ILC2s in mice (Fig. 1G) and upregulated *KLRG1* expression in active EoE patients’ esophagus-resident ILC2s compared with that in esophageal Th2 cells [12]. We analyzed 22 samples from patients with EoE and 10 from GERD patients (Fig. 1I, Supplementary Table 1). While GERD and EoE share chronic inflammation, they differ in their clinical symptoms, with GERD presenting with heartburn and EoE characterized by a narrowed esophagus structure [18]. Histologically, GERD patients exhibit increased lymphohistiocytic infiltration with basal cell hyperplasia, whereas EoE patients show marked eosinophilic infiltration (Supplementary Fig. 2A). The normal esophagus samples presented minimal immune cell presence, with only one of the 8 samples containing ILC2s (Fig. 1J, K), which is consistent with their low frequency in healthy mouse esophagi. Conversely, over half (13/22) of the esophageal tissues from EoE patients bore CD3e^−^KLRG1^+^ILC2s. Additionally, 3 out of 10 GERD patients presented with CD3e ^−^ KLRG1 ^+^ ILC2s (Fig. 1J, K and Supplementary Fig. 2B). These findings suggest that individuals with EoE are more likely to have ILC2s in their esophagus than are those with related esophageal diseases.

### Intranasal IL-33 induces esophageal eosinophilia, epithelial hyperplasia, and increased ILC2 numbers in mice

**Fig. 2:**
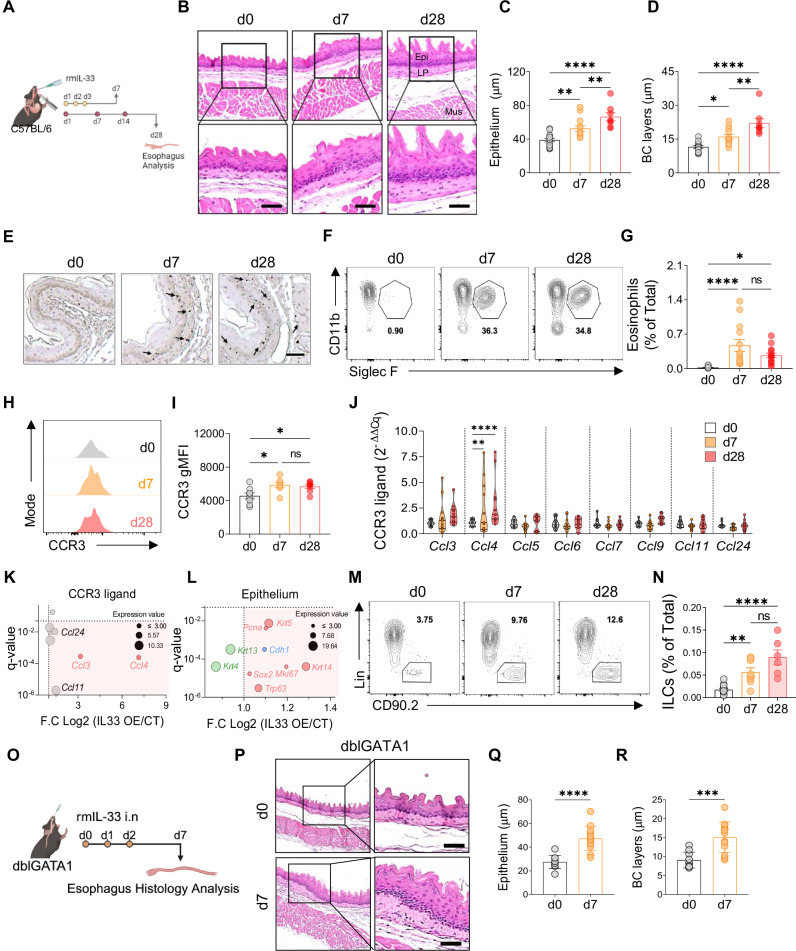
IL-33-induced EoE is characterized by eosinophilia, epithelial hyperplasia, and increased number of esophageal ILCs. **A** Schematic representation of acute and chronic EoE. **B** H&E-stained sections of control (d0), acute (d7), and chronic (d28) EoE mouse esophagi. Scale bars = 50 µm. (Epi: epithelium, LP: lamina propria, Mus: muscle). Quantification of epithelial thickness (**C**) and basal cell layer thickness (**D**) in EoE model mice. **E** Immunohistochemistry images of major basic protein 1 (MBP1)^+^ cells in EoE mouse esophagi. Flow cytometry plots showing eosinophils (**F**) and frequencies of esophageal eosinophils (**G**) in the acute and chronic EoE mouse models. Comparison of CCR3^+^ eosinophils in acute and chronic EoE. Histogram (**H**) and CCR3 gMFI from eosinophils (**I**). **J** Comparison of CCR3-associated chemokine gene expression in whole esophageal tissues with EoE. Analysis of CCR3-associated chemokine gene expression (**K**) and epithelium-associated gene expression (**L**) in IL-33-overexpressing mouse esophageal public RNA sequencing data. Flow cytometry plots (**M**) and frequencies (**N**) of esophageal-resident ILCs. **O** Schematic representation of acute EoE in eosinophil-deficient dblGATA1 mice. **P** Representative H&E-stained sections of control and acute EoE from dblGATA1 mice. Quantification of epithelial thickness (**Q**) and basal cell layer thickness (**R**) in dblGATA1 EoE model mice. All scale bars = 50 µm. The data were pooled from at least 2‒3 independent experiments and are presented as the means ± SEMs. *P ≤ 0.05, **P ≤ 0.01, ***P ≤ 0.001, ****P ≤ 0.0001, ns, not significant

A recent study demonstrated that epithelial-specific *Il33* gene overexpression leads to decreased body weight gain and esophageal edema [19]. Additionally, intranasal IL-33 induces robust esophageal eosinophilia, basal cells hyperplasia, and increased ILC2 numbers in mice, implicating IL-33 in EoE pathogenesis [20, 21]. Using an IL-33-induced murine EoE model, we aimed to elucidate the contribution of ILC2s, providing distinct insights compared with those of transgenic IL-33-overexpressing models. Acute and chronic EoE were generated by intranasal administration of rmIL-33 (Fig. 2A). Consistent with previous findings [19], we observed reduced weight gain (Supplementary Fig. 3A) and a significant increase in esophageal diameter in acute EoE mice treated with rmIL-33 (Supplementary Fig. 3B, C). Additionally, markedly increased esophageal epitheli and basal cell layer thickness, lamina propria thickening, and fibrosis were observed (Fig. 2B–D, Supplementary Fig. 3D). Immunohistochemistry and flow cytometry confirmed substantial eosinophil infiltration in rmIL-33-induced EoE mice (Fig. 2E–G), with elevated expression of CCR3 and *Ccl4*, indicating potential mechanisms of eosinophil recruitment (Fig. 2H–J).

Analysis of publicly available RNA sequencing data (GSE238122) further validated our model [19], with significant increases in *Ccl4* expression observed (Fig. 2K). Additionally, IL-33 overexpression in mice led to the upregulation of basal cell markers (*Krt14*, *Sox2*, and *Trp63*), epithelial markers (*Chd1*), and proliferative cell markers (*Mki67* and *Pcna*), along with decreased expression of differentiated epithelial markers (*Krt4* and *Krt13*) (Fig. 2L).

ILC2s, but not CD4^+^ T cells, presented an increased frequency within the esophagus following IL-33 administration, suggesting a specific role in EoE pathogenesis (Fig. 2M, N, Supplementary Fig. 3E). Investigation of chemokine receptors associated with ILC2s recruitment revealed no significant differences, indicating a distinct mechanism (Supplementary Fig. 3F). Further experiments using dblGATA1^–/–^ mice demonstrated that while eosinophils may influence certain aspects of IL-33-induced EoE, basal cell proliferation and epithelial thickening appear at least partially independent of eosinophils (Fig. 2O–R).

### Esophageal ILC2s accumulate at the epithelial border in EoE

**Fig. 3:**
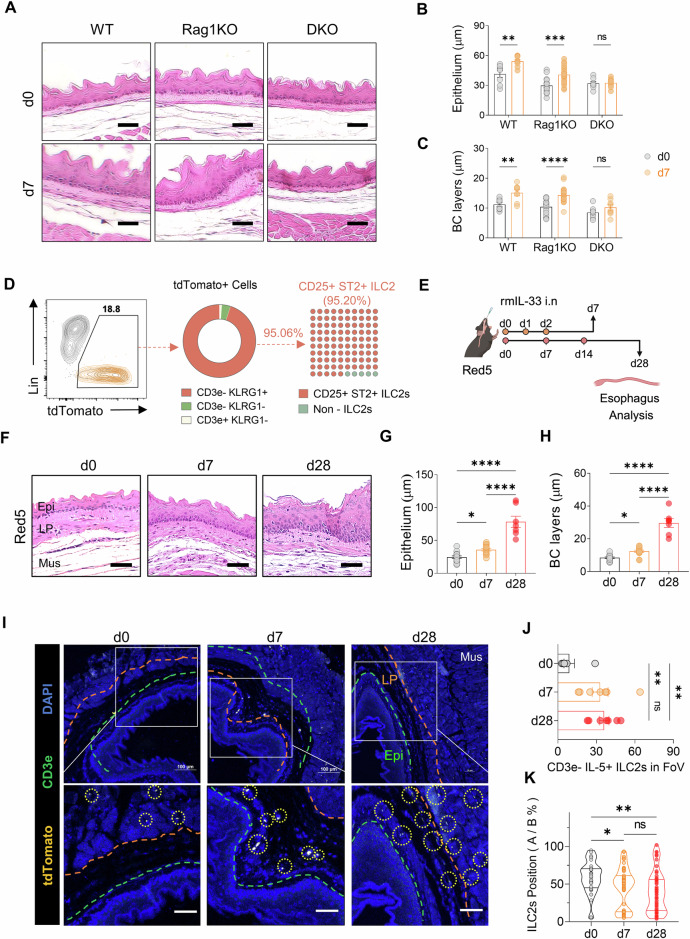
KLRG1^+^ ILC2 accumulation near the esophageal epithelium and promotion of epithelial thickening during EoE development. **A** H&E-stained sections of control and acute EoE from WT, Rag1KO, and DKO mice. Epithelial (**B**) and basal cell layer thickness (**C**) quantification in acute EoE model mice. **D** Flow cytometry analysis of tdTomato^+^ cells using ILC2 markers (KLRG1, CD25, and ST2) and T cells (CD3e) in Red5 (IL-5 tdTomato reporter, IL-5 deficient) mice. **E** Schematic representation of acute and chronic EoE in Red5 mice. **F** Representative H&E-stained sections showing control, acute, and chronic EoE in Red5 mice. Quantification of epithelial thickness (**G**) and basal layer thickness (**H**) in EoE-induced Red5 mice. **I** Immunofluorescence images of esophageal tdTomato^+^ immune cells in control, acute, and chronic EoE mice (yellow: IL-5 tdTomato; green: CD3e; blue: DAPI). The orange line denotes the boundary between the muscle and lamina propria; the green line indicates the boundary between the lamina propria and epithelium (Epi: epithelium; LP: lamina propria; Mus: muscle). **J** Quantification of CD3e^−^IL-5^+^ ILC2s in the region of uncropped original images (x200) of control, acute, and chronic EoE esophagus. **K** Localization of CD3e^−^IL-5^+^ ILC2s. All scale bars = 50 µm. The data were pooled from 2–3 independent experiments and are presented as the means ± SEMs. *P ≤ 0.05, **P ≤ 0.01, ***P ≤ 0.001, ****P ≤ 0.0001, ns, not significant

The accumulation of esophageal ILC2s at the epithelial border in EoE prompted us to hypothesize that type 2 inflammation triggered by ILC2s might drive epithelial alterations. Basal-cell hyperplasia, which is commonly associated with allergic respiratory disease, is correlated with the basal-cell production of alarmins such as IL-33 and TSLP, as well as with IL-4/IL-13-induced gene signaling in basal cells [22, 23]. To test this hypothesis, we induced acute EoE in WT and Rag1KO mice lacking T and B lymphocytes and Rag2/Il2rg-KO (DKO) mice lacking T and B cells and ILCs. While WT and Rag1KO mice presented with epithelial thickening and basal cell proliferation, DKO mice did not (Fig. 3A–C). A similar trend was observed for lamina propria thickening, a characteristic feature of EoE (Supplementary Fig. 4A, B), suggesting a pivotal role for ILC2s in IL-33-induced esophageal epithelial thickening.

To further investigate the role of ILC2s, we utilized IL-5 reporter mice (Red5), in which the tdTomato fluorescent reporter gene is inserted downstream of the *Il5* gene to visualize IL-5-expressing cells [24]. Flow cytometric analysis revealed that the majority of tdTomato-expressing CD45^+^ cells lacking a lineage marker were KLRG1^+^ILC2s (Fig. 3D). Induction of acute or chronic EoE in Red5 mice resulted in esophageal epithelial and basal-cell layer thickening similar to that in WT mice (Fig. 3E–H and Supplementary Fig. 4C, D). Immunofluorescence analysis confirmed the increased presence of CD3e^−^ tdTomato^+^ ILC2s in EoE sections compared with controls (Fig. 3I, J).

Given their role as tissue-resident cells, the spatial distribution of ILC2s is crucial for tissue repair and remodeling. We hypothesized that if ILC2s drive EoE-related epithelial thickening, they would be located closer to the epithelium. Indeed, activated KLRG1^+^ ILC2s were significantly closer to the basal-cell layer at days 7 and 28 post rmIL-33 challenge than on day 0 (Fig. 3K and Supplementary Fig. 4E), supporting the involvement of ILC2s in EoE-related epithelial changes.

### ILC2-derived Areg promotes esophageal basal cell proliferation

**Fig. 4:**
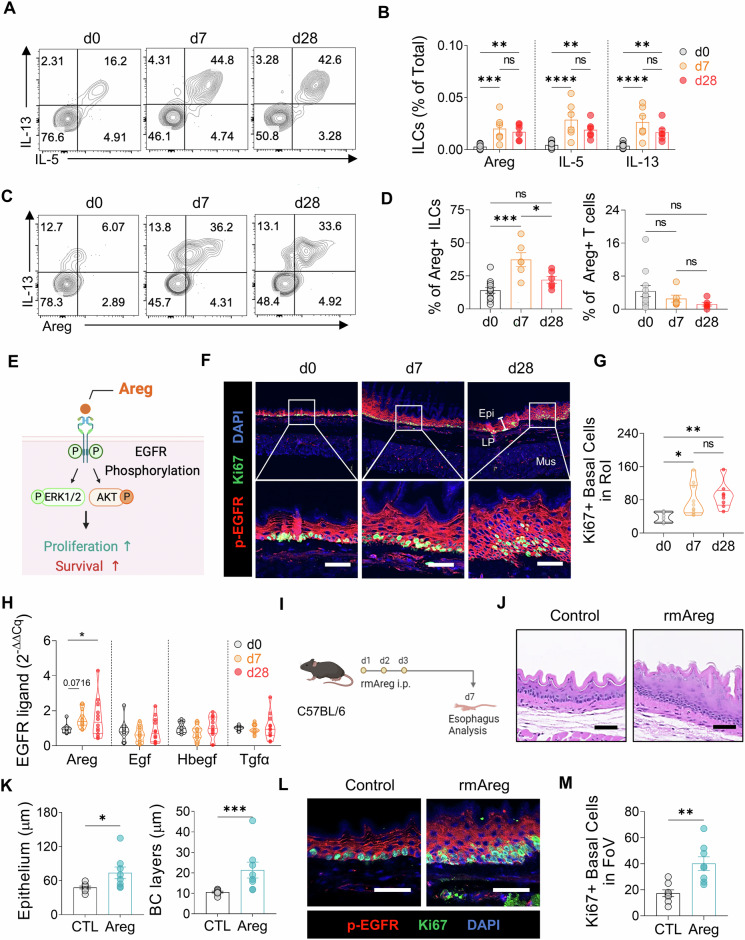
Areg expression by ILC2s in EoE development and its role in epithelial thickness and basal cell hyperplasia. **A** Representative flow cytometry plots of IL-5- and IL-13- producing esophageal ILCs. **B** Frequencies of IL-5- and IL-13-producing esophageal ILCs. **C** Flow cytometry analysis showing Areg production by esophageal ILCs. **D** Frequencies of Areg-producing esophageal ILCs (left) or CD4^+^ T cells (right). **E** Schematic illustration of the Areg-EGFR signaling cascade. **F** Immunofluorescence image analysis of the epithelium in the control, acute, and chronic EoE groups (red: p-EGFR; green: Ki67; blue: DAPI). (Epi: epithelium, LP: lamina propria, Mus: muscle). **G** Quantification of Ki67^+^ basal cells in the region of uncropped original images (magnification x200). **H** Comparison of the gene expression of EGFR ligands during EoE. **I** Schematic diagram of the intraperitoneal injection of recombinant murine Areg (rmAreg) in mice. **J** H&E-stained esophageal sections from control and rmAreg-treated mice. **K** Quantification of the esophageal epithelium (left) and basal cell layer (right) thickness in rmAreg-treated mice. **L** Immunofluorescence analysis of epithelial and basal cell hyperplasia in control and rmAreg-treated mice (red: p-EGFR; green: Ki67; blue: DAPI). **M** Quantification of Ki67^+^ basal cells in uncropped images of rmAreg-injected mice (magnification ×200). All scale bars = 50 µm. The data were compiled from at least 2‒3 independent experiments and are presented as the means ± SEMs. *P ≤ 0.05, **P ≤ 0.01, ***P ≤ 0.001, ****P ≤ 0.0001, ns, not significant

On the basis of our results, we hypothesized that esophageal ILC2s may contribute to the development of esophageal epithelial thickness and basal hyperplasia through their cytokine secretion. To address which specific ILC2 cytokines drive basal-cell hyperplasia in EoE, we conducted a flow cytometry analysis of esophageal immune cells in EoE, focusing on ILC2s expressing type 2 cytokines such as Areg, IL-5, and IL-13 (Fig. 4A–D). All type 2 cytokines from ILC2s are increased in EoE, but we focused on Areg for several reasons. First, IL-5 is unlikely to contribute to basal cell proliferation in EoE, as this proliferation persisted in IL-33-challenged Red5 mice, which lack functional IL-5 expression (Fig. 3F–H). Second, flow cytometry revealed elevated Areg-expressing esophageal ILC2s in IL-33-challenged mice, whereas Areg-expressing CD4^+^ T cells were nearly undetectable and did not change in frequency (Fig. 4D and Supplementary Fig. 5A–D). Moreover, gating on CD45^+^ Areg^+^ lymphocytes revealed that ILC2s are the dominant source of Areg in esophageal tissue, with minimal contributions from other lymphocytes, including T cells (Supplementary Fig. 5E, F).

Areg, which binds to EGFR and induces its tyrosine phosphorylation, triggers the ERK and AKT signaling cascades, leading to cell proliferation and epithelial-cell differentiation (Fig. 4E) [25]. Immunostaining of esophageal tissues revealed increased phospho-EGFR expression throughout the mucosa in EoE mice, along with increased proliferation of Ki67^+^ cuboidal basal cells (Fig. 4F, G, Supplementary Fig. 6A), indicating robust upregulation of phospho-EGFR in EoE mice model. Notably, quantitative RT‒PCR demonstrated that *Areg*, the sole EGFR ligand, was upregulated in EoE mice esophagi (Fig. 4H). Furthermore, the administration of rmAreg to WT mice without IL-33 induced abnormal epithelial thickening and basal cell proliferation, elevated phospho-EGFR levels, and increased the number of Ki67-expressing basal cells (Fig. 4I–M, Supplementary Fig. 6B, C), suggesting that ILC2-derived Areg play a role in promoting basal cell proliferation through EGFR phosphorylation in EoE. Thus, ILC2-derived Areg may contribute to epithelial remodeling in EoE by promoting basal cell proliferation through EGFR phosphorylation.

### Areg-induced EGFR phosphorylation promotes esophageal epithelial cell hyperplasia

**Fig. 5:**
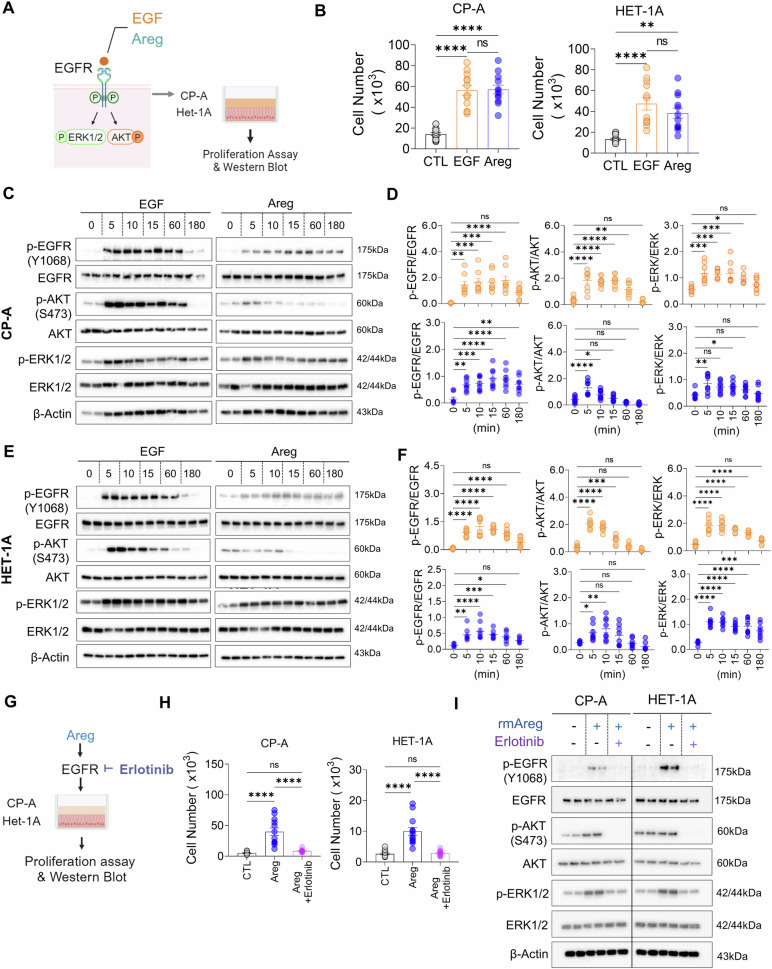
Areg-mediated stimulation of esophageal epithelial cell proliferation via the Areg-EGFR signaling pathway. **A** Schematic representation of the EGFR signaling pathway and the experimental design for EGF and Areg treatment of human esophageal epithelial cell lines (CP-A and HET-1A). **B** Total viable cell counts of CP-A and HET-1A cells treated with rmEGF (10 ng/ml) and rmAreg (100 ng/ml) for 3 days. **C**, **D** Western blotting (**C**) and densitometry quantification (**D**) of EGFR phosphorylation and downstream pathway activation induced by EGF and Areg in CP-A cell lines. **E**, **F** Western blotting (**E**) and densitometry quantification (**F**) of EGF- or Areg-treated HET-1A cells. **G** Experimental scheme for erlotinib (EGFR tyrosine kinase inhibitor) treatment of the CP-A and HET-1A cell lines. **H** Total viable counts of CP-A and HET-1A cells treated with rmAreg (100 ng/ml) for 3 days in the presence or absence of erlotinib (200 ng/ml). **I** Western blot analysis of EGFR phosphorylation and activation of downstream pathways (ERK1/2 and AKT) induced by Areg in CP-A and HET-1A cells treated with or without erlotinib. The data were pooled from at least 2‒3 independent experiments and are presented as the means ± SEMs. *P ≤ 0.05, **P ≤ 0.01, ***P ≤ 0.001, and ****P ≤ 0.0001; ns, not significant

Phosphorylation of EGFR initiates downstream signaling, including ERK1/2 and/or AKT [26]. Using the human esophageal epithelial cell lines CP-A and HET-1A, both of which express EGFR [27], we investigated the EGFR signaling pathways that drive Areg-induced hyperplasia in esophageal epithelial cells in EoE (Fig. 5A). Despite being squamous, CP-A is nontransformed, making it suitable for our study. Areg or EGF increased the number of cells equally (Fig. 5B), but Areg induced lower levels of phosphorylated EGFR, AKT, and ERK1/2 than did EGF, prolonging EGFR phosphorylation (Fig. 5C–F). These findings suggest that Areg-driven mitosis in esophageal epithelial cells may rely more strongly on EGFR signaling.

The significance of Areg-mediated EGFR signaling in esophageal epithelial cell proliferation was confirmed by treating cell lines with erlotinib, an EGFR tyrosine kinase inhibitor (Fig. 5G) [28]. Erlotinib completely inhibited Areg-induced cell proliferation (Fig. 5H) and blocked Areg-induced phosphorylation of EGFR, AKT, and ERK1/2 (Fig. 5I).

### Blocking Areg-EGFR signaling reduces esophageal hyperplasia and inflammation in the EoE mouse model

**Fig. 6:**
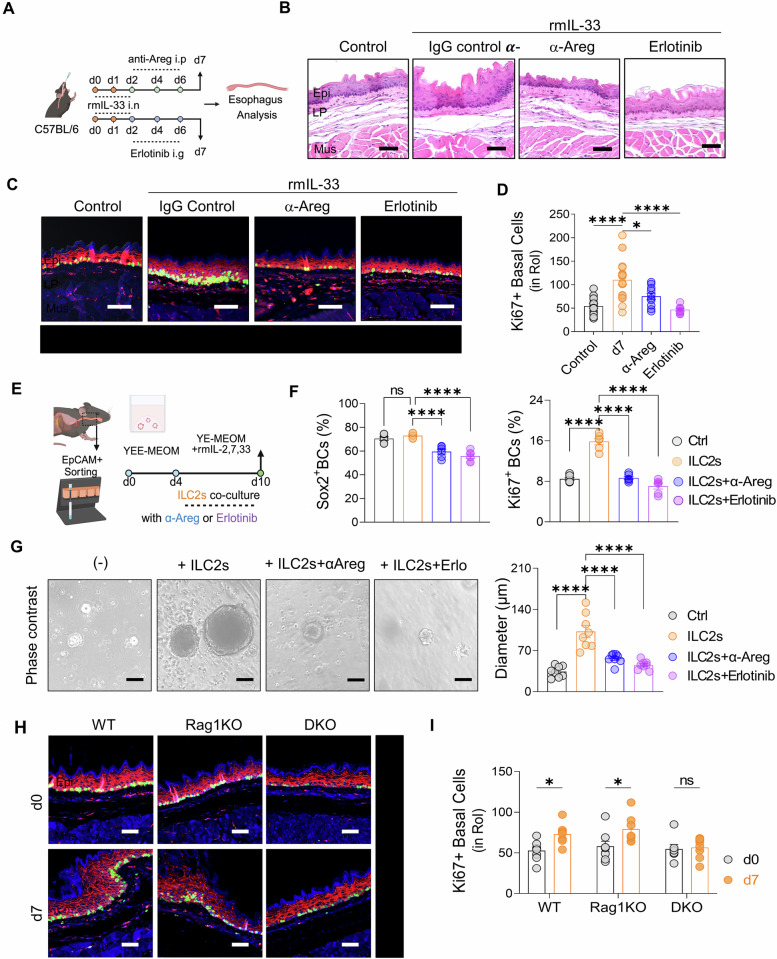
Epithelial remodeling and inflammation in EoE are attenuated by blockade of Areg-EGFR signaling. **A** Experimental design for the in vivo administration of αAreg and erlotinib to C57BL/6 mice with acute EoE. **B** H&E-stained sections showing the esophagus of control and acute EoE mice treated with or without αAreg and erlotinib. **C** Immunofluorescence image analysis of the esophageal epithelium and basal cell hyperplasia in EoE mice following EGFR signaling blockade (red: p-EGFR; green: Ki67; blue: DAPI, X200). **D** Quantification of Ki67^+^ basal cells in the uncropped original images of (**C**). **E** Schematic representation of mouse esophageal organoid and ILC2 coculture. **F** Frequencies of Sox2^+^ and Ki67^+^ basal cells. **G** Representative phase contrast images of the esophageal epithelial organoids in each group, along with quantification of organoid diameter. Scale bars = 50 µm. **H** Immunofluorescence image analysis of the esophageal epithelium and basal cell hyperplasia in EoE from WT, Rag1KO, and DKO mice (red: p-EGFR; green: Ki67; blue: DAPI, X200). **I** Quantification of Ki67^+^ basal cells in the uncropped original images of (**H**). The data were pooled from at least 2‒3 independent experiments and are presented as the means ± SEMs. *P ≤ 0.05, **P ≤ 0.01, ***P ≤ 0.001, and ****P ≤ 0.0001; ns, not significant

To validate the involvement of the Areg-EGFR axis in EoE pathogenesis, we used erlotinib to target this axis. In the acute-EoE murine model, the mice received either an Areg-neutralizing antibody or erlotinib on days 2, 4, and 6 (Fig. 6A). Both treatments significantly reduced esophageal epithelial hyperplasia and immune cell infiltration (Fig. 6B and Supplementary Fig. 7A–C), as well as thickening of the esophageal lamina propria (Supplementary Fig. 7E). A reduction in eosinophil numbers might correlate with decreased tissue expression of eosinophil chemokines, such as *Ccl4* (Supplementary Fig. 7D). Additionally, mice treated with the anti-Areg antibody and erlotinib presented decreased numbers of phospho-EGFR-expressing esophageal epithelial cells and Ki-67^+^ proliferating stem-like basal cells (Fig. 6C, D).

To model the effects of ILC2s on esophageal epithelial cells, we cocultured primary esophageal basal cells (EpCAM^+^ Sox2^+^) with ILC2s in 3D organoids (Fig. 6E, Supplementary Fig. 8A). This coculture significantly enhanced basal cell proliferation and organoid growth, whereas blocking Areg reduced stemness, proliferation, and growth. Additionally, the EGFR inhibitor erlotinib suppressed both organoid growth and basal cell proliferation, confirming that ILC2-derived Aregs act through EGFR signaling (Fig. 6F, G and Supplementary Fig. 8B).

These in vitro results were further corroborated in vivo, where the pivotal role of ILC2s in initiating the Areg-EGFR axis and driving esophageal hyperplasia was demonstrated in WT, Rag1KO, and DKO mice. The increased phospho-EGFR^+^ epithelial area and Ki-67^+^ basal cells observed in WT and Rag1KO mice were significantly diminished in DKO mice lacking ILCs (Fig. 6H, I and Supplementary Fig. 7F), supporting the critical role of the ILC2-Areg-EGFR pathway in EoE pathogenesis. In conclusion, our findings demonstrate that the ILC2-derived Areg-EGFR axis plays a pivotal role in driving esophageal epithelial hyperplasia and immune cell infiltration in EoE and that targeting this pathway with erlotinib or Areg-neutralizing antibodies effectively reduces these pathological features.

### ILC2-derived amphiregulin in human EoE patients

**Fig. 7:**
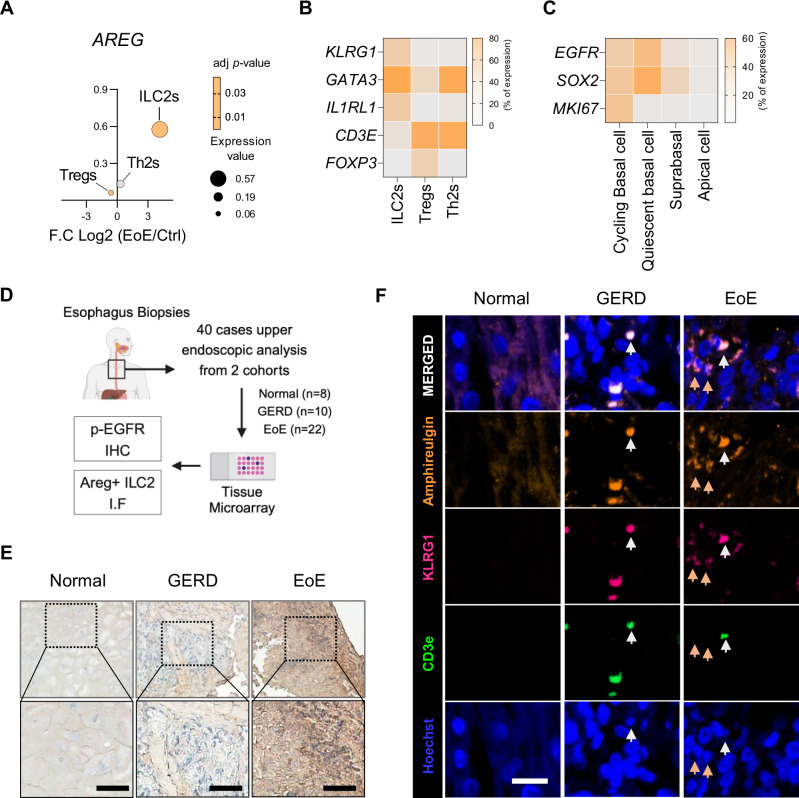
Amphiregulin from ILC2s in human EoE patients. **A** Differential expression of *AREG* among ILC2s, Tregs, and Th2 cells, with adjusted *P* values indicated by the dot color, expression levels represented by the dot size (y-axis), and fold change shown on the x-axis. **B** Proportions of cells expressing key markers (*KLRG1, GATA3, and IL1RL1*) across different immune cell types in patients with EoE. **C** Proportion of epithelial cells expressing marker genes in EoE patients. All processed gene count matrices and embeddings were derived from EoE patient data (healthy: *n* = 12; remission: *n* = 11; active: *n* = 14). **D** Study design for human esophageal biopsies, including healthy controls (*n* = 6), GERD patients (*n* = 10), and EoE patients (*n* = 22) from two cohorts. **E** Representative immunohistochemistry analysis of p-EGFR (blue: nuclei; DAB brown: p-EGFR). Scale bar = 50 µm. **F** Immunofluorescence images from healthy controls, GERD patients, and EoE patients (Amphiregulin: Orange; KLRG1: Magenta; CD3e: Green; Hoechst: Blue). The green arrows indicate CD3^+^ T cells, whereas the orange arrows indicate KLRG1^+^ ILC2s. Scale bar = 20 µm. The data were pooled from at least 2‒3 independent experiments and are presented as the means ± SEMs. *P ≤ 0.05, **P ≤ 0.01, ***P ≤ 0.001, and ****P ≤ 0.0001; ns, not significant

To validate our findings in a human context, we analyzed both public single-cell RNA sequencing data from human EoE patients [12] and esophageal biopsy samples from our own patient cohort. Single-cell RNA-seq data revealed that ILC2s presented significantly elevated *AREG* expression compared with other immune cells, such as Tregs and Th2 cells (Fig. 7A). Additionally, key ILC2 markers, including *KLRG1*, *GATA3*, and *IL1RL1* (the receptor for IL-33), were highly upregulated in esophageal-resident ILC2s (Fig. 7B). The epithelial cells of these patients also expressed critical genes associated with ILC2-derived amphiregulin signaling (Fig. 7C).

To corroborate these transcriptomic findings at the protein level, we analyzed esophageal biopsies from our cohort, which included patients with EoE or GERD and healthy controls (Fig. 7D). Immunohistochemistry revealed significantly increased p-EGFR expression in the esophageal epithelium of EoE patients compared with that of GERD patients and healthy controls (Fig. 7E). Immunofluorescence analysis further confirmed elevated levels of amphiregulin and the presence of KLRG1^+^ ILC2s in EoE tissues, alongside CD3^+^ T cells (Fig. 7F). These results suggest that IL-33-induced EoE is directly dependent on Areg produced by esophageal ILC2s, which activate EGFR in epithelial cells, including basal cells, in both mice and humans, leading to abnormal proliferation, layer formation, and epidermal thickening.

## Discussion

The integrity of the esophageal epithelium is maintained through continuous regeneration driven by epithelial stem cells, ensuring protection against external insults during food passage [6, 29]. Disruptions in this homeostatic process, particularly basal-cell hyperplasia, characterize EoE, highlighting immune dysregulation [30]. Our study underscores a pivotal role for ILC2s in this process, demonstrating the presence of KLRG1^+^ ILC2s in the esophagus of active EoE patients and their ability to produce the EGF family member Areg, which directly stimulates basal-cell proliferation in a murine EoE model. These findings shed new light on the pathogenic roles of ILC2s in the upper gastrointestinal tract.

Clinical investigations into EoE underscore the crucial involvement of ILC2s in disease pathogenesis. Studies have revealed increased numbers of ILC2s in the esophagus of active pediatric EoE patients compared with healthy controls, alongside increased levels of IL-33, a key activator of ILC2s, and IL-13, a cytokine produced by ILC2s [11, 31]. Moreover, the eosinophilic inflammation characteristic of EoE is attributed to the high production of type 2 cytokines, including IL-5 and IL-13, by ILC2s [24]. These cytokines contribute to tissue remodeling, impacting barrier function and cellular renewal in the esophagus [23, 32]. Our findings align with these observations, showing that ILC2s and their cytokine Areg play a central role in EoE development. Inhibition of ILC2s and Areg leads to a reduction in basal-cell hyperplasia and epithelial thickening in murine models, implicating a cascade initiated by epithelial injury, IL-33 release, and subsequent ILC2 activation, ultimately resulting in Areg-mediated basal-cell proliferation.

This hypothesis is indeed supported by our study. We observed a greater prevalence of ILC2s in the esophagus of EoE patients than in that of healthy or GERD patients (59% vs. 10% or 33%, respectively). Additionally, intranasal IL-33 challenge upregulated ILC2s in the esophagus, inducing hallmark EoE characteristics such as basal-cell proliferation, epithelial thickening, and eosinophilia. The absence of ILCs or anti-Areg antibody treatment inhibits EoE development, whereas intraperitoneal injection of rmAreg recapitulates basal cell proliferation and epithelial thickening. These findings support the notion that EoE may be initiated by epithelial cell injury, triggering IL-33 release, subsequent ILC2 activation, and Areg-mediated basal cell proliferation.

Furthermore, our observations in the normal murine esophagus revealed that ILCs were the predominant lymphocyte population, with T cells being rare. Among ILC subtypes, ILC2s constitute the majority (90%) in the homeostatic esophagus and express higher levels of KLRG1 than do lung ILC2s. Previous studies have indicated that KLRG1-expressing ILCs in the lung are inflammatory ILC2s that secrete relatively high levels of cytokines [33, 34]. Consistently, we found that KLRG1^+^ ILC2s in the esophagus secreted more type 2 cytokines than did lung ILC2s. The predominance of ILCs over T cells in the esophagus, in contrast to those in the lung, suggests potential functional differences between ILC populations in different tissues.

Our observations concerning ILC2 relocation and its contribution to detrimental epithelial remodeling in EoE are consistent with those of previous studies [12]. In our IL-33-induced EoE mouse model, we observed the relocation of ILC2s from the muscular layer of the normal esophagus to the lamina propria, where they formed clusters near the basal-cell layer following EoE induction. This observation supports the hypothesis that ILC2s, rather than eosinophils, directly contribute to the detrimental epithelial remodeling associated with EoE.

Therapeutic strategies for ILC-mediated diseases can target upstream signals that activate ILCs, the ILCs themselves, or their cytokines [35]. In our study, murine EoE was associated with elevated production of not only Areg but also IL-5 and IL-13 by ILC2s. However, among these, Areg is the most promising therapeutic target for several reasons. First, IL-5 was unlikely to play a key role in EoE epithelial thickening, as this feature remained fully present in the IL-5-deficient Red5 mice. Second, previous research has highlighted the protective role of ILC2-derived Areg in preventing lung pathogenicity and promoting epithelial-cell hyperplasia [14]. Additionally, Areg, a member of the EGF family, induces the proliferation and differentiation of normal epithelial cells by binding to EGFR on these cells [25, 36]. Notably, Areg emerged as the sole EGFR-binding factor expressed in the esophagus of patients with EoE in our study. Our investigation further supported the critical role of Areg in EoE pathogenesis. We observed EGFR activation in esophageal epithelial cells in murine EoE and healthy mice following rmAreg injection, which also recapitulated the basal cell proliferation and epithelial thickening observed in EoE. Moreover, IL-33-challenged mice lacking ILCs did not exhibit EGFR activation or basal cell proliferation. Thus, ILC2-derived Aregs are crucial factors in promoting esophageal epithelial thickening in EoE.

Building on these observations, we explored the potential therapeutic implications. Treatment with anti-Areg or erlotinib, an inhibitor of EGFR signaling, effectively blocked the epithelial thickening observed in IL-33-challenged mice. Notably, these inhibitors have been extensively studied in head and neck cancer and esophagogastric cancer, effectively targeting abnormal epithelial cell proliferation [37, 38]. Additionally, ILC2-derived IL-5 plays a crucial role in eosinophil survival and recruitment, while Aregs can be directly reprogrammed into activated eosinophils [39, 40]. Although our study focused primarily on the role of ILC2s in EoE pathogenesis, CD4^+^ T cells are also key contributors, particularly in allergen-driven inflammation [41]. Interestingly, the administration of Areg-EGFR signaling inhibitors blocked eosinophilic features in the esophagus, even in the absence of ILCs. Therefore, our findings suggest that epithelial-cell hyperplasia in EoE may involve the release of Areg by ILC2s and its binding to EGFR on basal cells. Therefore, Areg-EGFR inhibitors or targeting ILC2s may represent promising therapeutic options for treating EoE.

In conclusion, our study revealed the previously overlooked role of KLRG1^+^ ILC2s in maintaining esophageal homeostasis and their significant involvement in the pathogenesis of EoE. Through our investigations, we elucidated how these cells contribute to basal-cell hyperplasia and mucosal thickening via the Areg-EGFR axis. These findings not only enhance our understanding of the complex interplay between innate immunity and esophageal health but also provide a solid basis for the development of targeted therapeutic interventions. Further elucidation of these underlying mechanisms in future research holds promise for advancing the management of EoE and related conditions.

## Materials and methods

### Mice

Six- to eight-week-old C57BL/6 J mice were purchased from Koatech (Gyeonggi-do, South Korea). B6.129S7-Rag1^tm1Mom^/J (Rag1KO) mice, B6(C)-Il5^tm1.1(icre)Lky^/J (IL-5 tdTomato reporter [Red5] and IL-5 deficient) mice, and C.129S1(B6)-Gata1^tm6Sho^/J (dblGATA1) mice, which lack eosinophils, were purchased from Jackson Laboratories (Bar Harbor, ME, USA). C57BL/6NTac. Cg-Rag^2tm1^Fwa Il2rg^tm1Wjl^ (Rag2^–/–^ IL2rg^–/–^ double-KO [DKO]) mice were purchased from Taconic (Germantown, NY, USA). All animals were housed in the SPF animal care facility at Seoul National University Hospital (SNUH). All murine studies were performed at the Research Institute of SNUH, which is accredited by AAALAC International. The murine experiments were also approved by the IACUC of SNUH Biomedical Research Institute (IACUC, # 35-2019-0135).

### Animal experiments

Recombinant mouse IL-33 (rmIL-33; BioLegend, 580508) was used to induce EoE in the mice. A 250 ng dose of rmIL-33 per mouse was instilled intranasally into lightly anesthetized mice. To mimic acute and chronic EoE, the mice were administered IL-33 on days 0, 1, and 2 and days 0, 7, and 14, respectively, and were sacrificed on days 7 and 28 after the initial injection. Control IgG (R&D Systems) or recombinant mouse Areg (rmAreg; R&D systems, 989-AR-100/CF) was administered intraperitoneally (2 µg per mouse) to wild-type C57BL/6 J mice on days 0, 1, and 2, and the mice were sacrificed on day 7. Goat IgG control (R&D Systems, AB-108-C), anti-Areg (R&D Systems, AF989) intraperitoneal injection or the EGFR tyrosine kinase inhibitor erlotinib hydrochloride (MCE, HY12008) via oral gavage (10 mg/kg) were used on days 2, 4, and 6. The rats were sacrificed on day 7.

### Mouse histology, immunohistochemistry, and immunofluorescence image analysis

All H&E-stained images and IHC images were captured via an Olympus IX53 microscope (Center Valley, PA, USA). To analyze esophageal histology, 3 randomly selected regions of each mouse esophagus were captured at 200X magnification and quantified via ImageJ software (NIH, MD, USA). Esophageal epithelium thickness was measured by drawing 3 straight lines from the bottom to the top of the esophageal epithelium and obtaining the average length. The basal cell layer thickness was measured by drawing 3 straight lines from the basal membrane to the end of unstratified basal cells in the basal cell layer. Lamina-propria thickness was measured by drawing 3 straight lines from the top to the bottom of the lamina propria.

To investigate eosinophil infiltration, we used immunohistochemistry image analysis. The antibodies and IHC staining kits used for staining were as follows: Rb-anti-M MBP1 (ab187523) and TripleStain IHC Kit: M&R&Rt on rodent tissue (DAB, HRP/Green & AP/Red) (ab183297).

To investigate basal-cell hyperplasia and IL-5^+^ ILC2 accumulation in the esophageal mucosa of mice, we used immunofluorescence image analysis. The antibodies used for staining were as follows: Rb-anti-M/H p-EGFR (Y1068) (1:200; Abcam, ab40815), Rat-anti-M/H Ki67 (1:200; Invitrogen, 14-5698-82), and Rat-anti-M/H CD3 (1:200; Abcam, ab11089). Primary antibody-labeled sections were incubated with the secondary antibodies Dk-anti-Rb AF594 (1:500; Invitrogen, A-21207) or Dk-anti-Rat AF488 (1:500; Invitrogen, A-21208). One drop of ProLong diamond antifade mountant with DAPI (Invitrogen, P36961) was added, images were acquired via Nikon confocal A1 microscopy (Nikon, Tokyo, Japan), and the analysis was performed via the Nikon NIS-Elements Viewer (Nikon, Tokyo, Japan).

### Immune cell preparations

The esophagus was incubated in RPMI 1640 with 2 mM EDTA (Sigma, E7889-100 ml) at 37 °C for 20 min and then minced. The minced tissue was then digested with collagenase type 4 (Worthington, LS004189) and DNase1 (Sigma, DN25-1G) in RPMI 1640 at 37 °C for 1 h. The single-cell suspension was filtered through a 40 µm cell strainer, and red blood cells were removed by treating the cells with RBC lysis buffer (BioLegend, 420301).

For single-cell isolation, mechanically minced lung was digested with collagenase type 4 and DNase 1 in RPMI 1640 at 37 °C for 1.5 h. The resulting single-cell suspension was filtered through a 40 µm cell strainer, and red blood cells were removed as described above.

### Flow cytometry analysis

Single cells were suspended in cold PBS, stained with the Zombie Aqua Fixable Viability Kit (BioLegend, 423102) and treated with a mouse CD16/32 FC blocking antibody (BioLegend, 156604). To assess cytokine production, single cells were restimulated with 100 ng/ml PMA (Sigma, P8139-1MG), 1 µg/ml ionomycin (Sigma, I0634-1MG), and 0.7 µl/ml Golgi stop (BD biosciences, 554715) for 3 h. Single cells were then fixed and permeabilized via a fixation/permeabilization solution kit (BD biosciences, 554715) and a Foxp3 transcription factor staining buffer set (Invitrogen, 00-5523-00). The absolute numbers of cultured ILC2s were calculated with Precision Count Beads™ (BioLegend, 424902) according to the manufacturer’s instructions. All flow cytometry experiments were performed via a BD LSR Fortessa^TM^ X-20 (BD, NJ, USA) and analyzed via FlowJo (V10) software (BD, NJ, USA). The mouse antibodies used for flow cytometry included anti-CD45 (30-F11), anti-CD19 (1D3), anti-CD49b (DX5), anti-F4/80 (BM8), anti-FcεRIα (MAR-1), anti-CD127 (A7R34), anti-CD90.2 (30-H12), anti-CD25 (PC61), anti-IFN-γ (XMG1.2), anti-IL-17A (TC11-18H10.1), anti-IL-5 (TRFK5), anti-ICOS (C398.4 A), anti-KLRG1 (2F1), anti-SCA1 (D7), PerCP-Cy5.5-streptavidin (405214), anti-CCR3 (J073E5), anti-CCR9 (9B1), anti-CCR7 (4B12), anti-CCR4 (2G12), anti-CCR6 (29-2L17), anti-SiglecF (S17007L), anti-Gata3 (L50-823), anti-Tbet (4B10), and anti-Rorγt (B2D). Additional antibody information can be found in supplemental Table 2.

### Culture of the CP-A and HET-1A human esophageal epithelial cell lines

The hTERT-immortalized human Barrett’s esophagus epithelial cell lines CP-A (ATCC, KR-42421) and HET-1A (ATCC, CRL-2692) were employed, and subculture and subculture and handling procedures were followed with the ATCC cell line culture manufacturer in the BEGM BulletKit (Lonza, CC-3170) (BEBM: Lonza, CC-3171 and BEGM™ Bronchial Epithelial Cell Growth Medium SingleQuots™ Supplements and Growth Factors: Lonza, CC-4175). The CP-A cell line was established from an area of nondysplastic metaplasia, and HET-1A was isolated from the normal esophagus. To quantify cell proliferation, CP-A and HET-1A cell lines were treated with recombinant mouse EGF (rmEGF; 10 ng/ml; Corning, CB-40001), rmAreg (100 ng/ml), and erlotinib (200 ng/ml) in BEBM (Lonza, CC-3171) for 72 h. The numbers of CP-A and HET-1A cells were subsequently quantified.

### Western blot

CP-A and HET-1A were starved overnight in fresh BEBM and then treated with 100 ng/ml Areg (R&D systems, 989-AR-100/CF) or 10 ng/ml EGF (R&D systems, 2028-EG-200) in BEBM for 5, 10, 15, 60, and 180 min. To verify that Areg induces the EGFR signaling pathway, starved CP-A and HET-1A cells were challenged with 100 ng/ml rmAreg for 10 min with or without 200 ng/ml erlotinib hydrochloride (MCE, HY12008).

The CP-A and HET-1A cells were lysed and prepared for western blot analysis. The primary antibodies used for immunoblotting were as follows: rabbit anti-H/M beta-actin (1:2000, Thermo Fisher Scientific, BS-0061R), Rb anti-H/M p-EGFR (Y1068) (1:1000, Cell Signaling Technology, 2234S), Rb anti-H/M EGFR (1:1000, Cell Signaling Technology, 4267S), Rb anti-H/M p-AKT (S473) (1:1000, Cell Signaling Technology, 4060S), Rb anti-H/M AKT (1:1000, Cell Signaling Technology, 9272S), Rb anti-H/M p-ERK1/2 (1:1000, Cell Signaling Technology, 9101S), and Rb anti-H/M ERK1/2 (1:1000, Cell Signaling Technology, 9102S). Anti-Rb HRP was used as the secondary antibody (1:2000, Thermo Fisher Scientific, 7074P2). All densitometric analyses were performed via ImageJ (USA, NIH).

### Quantitative real-time PCR

Mouse whole esophageal tissues were homogenized with TRIzol (Invitrogen, USA) Reagent. cDNA was synthesized via a SeniFAST cDNA synthesis kit (Bioline, UK). qPCR was performed with a SeniFAST SYBR Lo-ROX kit (Bioline, UK) or Probe Lo-ROX kit (Bioline, UK) with a CFX96 Real-Time PCR Detection System (Bio-Rad, California, USA) according to the manufacturer’s instructions. All primers were obtained from Integrated DNA Technologies (IdT; Iowa, USA). The relative expression of the target genes was normalized to that of Gapdh via the 2^-*ΔΔ*CT^ method.

### Isolation of mouse primary esophagus basal cells and 3D organoid formation

Esophageal epithelial cells were successfully isolated from C57BL/6 J mice *via* EpCAM^+^ microbeads following enzymatic dissociation with TrypLE Express. Single-cell suspensions displayed high viability and purity, as confirmed by flow cytometry. The seeded cells (2 × 10³ cells per well) embedded in Matrigel formed well-structured organoids by day 4 of culture in YEE-MEOM medium. The organoids were spherical in shape, with diameters exceeding 20 µm. E-MEOM facilitated efficient organoid growth, and the Matrigel droplets remained intact throughout the culture period.

### ILC2 coculture with esophageal organoids

Lung-derived ILC2s were cocultured with esophageal organoids in YE-MEOM supplemented with IL-2, IL-7, and IL-33, along with either anti-Areg (100 ng/mL) or erlotinib (10 µM). The coculture conditions supported the continued expansion and viability of both ILC2s and organoids over the 6-day period. Organoid size increased notably in the presence of ILC2s, with significant differences observed between the anti-Areg and erlotinib treatment groups. On day 6, organoid diameters were measured, and those cocultured with ILC2s in anti-Areg media presented an average diameter increase of 25% compared with that of the control conditions (p < 0.05). Conversely, the organoids treated with erlotinib were smaller than those in both the control and anti-Areg groups were, suggesting that EGFR inhibition adversely affected organoid growth in the ILC2 coculture system. Organoid size quantification was performed by measuring the diameter under an Olympus IX53 microscope.

### Studies on human samples

Tissue microarrays (TMAs) were obtained from human esophageal tissues from two cohorts of patients. The first cohort included normal tissues from healthy controls (*n* = 8), esophageal mucosa tissues from patients with GERD (*n* = 10), and esophageal mucosa tissues from patients with EoE (*n* = 22) at Jeju National University Hospital and Hanyang University Hospital, South Korea (Supplementary Table 1). This study was approved by the Jeju University Hospital Institutional Review Board (IRB number 2020-11-003). All patients consented in writing to the use of their samples for scientific research.

The primary antibodies used for immunofluorescence image analysis were as follows: Rb-anti-H amphiregulin (1:100, Abcam, ab234750), Rat-anti-M/H CD3e (1:200, Abcam, ab11089) and PerCP-Cy5.5 M/H KLRG1 (1:200, Biolegend, 138418). The primary antibody-labeled sections were then incubated with the secondary antibodies DK-anti-Rat AF488 (1:500; Invitrogen, A-21208) and Dk-anti-Rb AF594 (1:500; Invitrogen, A-21207). Nuclei were stained with Hoechst (1:2000, Invitrogen, H3570) and mounted with VECTASHIELD® Antifade Mounting Medium (Vector Lab, H-1000-10). Images were acquired via Nikon confocal A1 microscopy.

To investigate p-EGFR expression in human esophageal samples, we used immunohistochemistry image analysis. The antibodies and IHC staining kits used for staining were as follows: Rb-anti-p-EGFR (Cell Signaling Technology, 2234S) and TripleStain IHC Kit: M&R&Rt on rodent tissue (DAB, HRP/Green & AP/Red) (ab183297). All the IHC images were captured via an Olympus IX53 microscope (Center Valley, PA, USA).

### Analysis of public bulk mRNA-seq data and single-cell RNA-seq data

For validation of our EoE mouse model, we selected a public IL-33-overexpressing (IL33 OE) mouse esophagus dataset, which was obtained from Masuda et al. (GSE238122) and generated via the Illumina HiSeq platform. The differentially expressed genes (DEGs) with an adjusted *q* < 0.05 were considered statistically significant, and a multiple variable plot was generated via log2 FC, adjusted q-scores and *log2* RNA expression *values to* visualize the DEGs.

To determine the clinical importance of ILC2-derived AREG in human EoE, we utilized public single-cell RNA-seq data from human EoE patients provided by Jiarui Ding et al. (Single Cell Portal SCP1242) [12]. Processed gene count matrices and embeddings were examined to explore AREG expression and its associated pathways in the context of EoE.

### Statistical analysis

All the statistical analyses were performed via GraphPad Prism 9.5.1 software (Graph Pad, La Jolla, CA, USA). For comparisons of statistical significance, the Mann‒Whitney U test or one-way ANOVA with post hoc Tukey’s multiple comparisons test was used. For comparisons involving more than two datasets, two-way ANOVA was used. The data are presented as the mean ± standard error of the mean (SEM), and p values less than 0.05 were considered statistically significant. The numbers of samples per group and replicate experiments are indicated in the figure legends.

## Supplementary information

Supplementary figure and tables

## Data Availability

All data supporting this study are included within the article and/or supplementary materials.

## References

[1] Dellon ES, Hirano I. Epidemiology and natural history of eosinophilic esophagitis. Gastroenterology. 2018;154:319–32.e3. 28774845 10.1053/j.gastro.2017.06.067 PMC5794619

[2] Muir A, Falk GW. Eosinophilic esophagitis: a review. JAMA. 2021;326:1310–8. 34609446 10.1001/jama.2021.14920 PMC9045493

[3] Mulder DJ, Justinich CJ. Understanding eosinophilic esophagitis: the cellular and molecular mechanisms of an emerging disease. Mucosal Immunol. 2011;4:139–47. 21228772 10.1038/mi.2010.88

[4] Davis BP, Stucke EM, Khorki ME, Litosh VA, Rymer JK, Rochman M, et al. Eosinophilic esophagitis-linked calpain 14 is an IL-13-induced protease that mediates esophageal epithelial barrier impairment. JCI Insight. 2016;1:e86355. 27158675 10.1172/jci.insight.86355 PMC4855700

[5] DeWard AD, Cramer J, Lagasse E. Cellular heterogeneity in the mouse esophagus implicates the presence of a nonquiescent epithelial stem cell population. Cell Rep. 2014;9:701–11. 25373907 10.1016/j.celrep.2014.09.027 PMC4223874

[6] Zhang Y, Jiang M, Kim E, Lin S, Liu K, Lan X, et al. Development and stem cells of the esophagus. Semin Cell Dev Biol. 2017;66:25–35. 28007661 10.1016/j.semcdb.2016.12.008 PMC5474349

[7] Steiner SJ. Severity of basal cell hyperplasia differs in reflux versus eosinophilic esophagitis. J Pediatr Gastroenterol Nutr. 2006;42:506–9. 16707971 10.1097/01.mpg.0000221906.06899.1b

[8] Ghaedi M, Takei F. Innate lymphoid cell development. J Allergy Clin Immunol. 2021;147:1549–60. 33965092 10.1016/j.jaci.2021.03.009

[9] Meininger I, Carrasco A, Rao A, Soini T, Kokkinou E, Mjosberg J. Tissue-specific features of innate lymphoid cells. Trends Immunol. 2020;41:902–17. 32917510 10.1016/j.it.2020.08.009

[10] Ham J, Shin JW, Ko BC, Kim HY. Targeting the epithelium-derived innate cytokines: from bench to bedside. Immune Netw. 2022;22:e11. 35291657 10.4110/in.2022.22.e11 PMC8901708

[11] Doherty TA, Baum R, Newbury RO, Yang T, Dohil R, Aquino M, et al. Group 2 innate lymphocytes (ILC2) are enriched in active eosinophilic esophagitis. J Allergy Clin Immunol. 2015;136:792–4.e3. 26233928 10.1016/j.jaci.2015.05.048 PMC4562810

[12] Ding J, Garber JJ, Uchida A, Lefkovith A, Carter GT, Vimalathas P, et al. An esophagus cell atlas reveals dynamic rewiring during active eosinophilic esophagitis and remission. Nat Commun. 2024;15:3344. 38637492 10.1038/s41467-024-47647-0 PMC11026436

[13] Berasain C, Avila MA. Amphiregulin. Semin Cell Dev Biol. 2014;28:31–41. 24463227 10.1016/j.semcdb.2014.01.005

[14] Monticelli LA, Sonnenberg GF, Abt MC, Alenghat T, Ziegler CGK, Doering TA, et al. Innate lymphoid cells promote lung-tissue homeostasis after infection with influenza virus. Nat Immunol. 2011;12:1045–54. 21946417 10.1031/ni.2131 PMC3320042

[15] Monticelli LA, Osborne LC, Noti M, Tran SV, Zaiss DM, Artis D. IL-33 promotes an innate immune pathway of intestinal tissue protection dependent on amphiregulin-EGFR interactions. Proc Natl Acad Sci USA. 2015;112:10762–7. 26243875 10.1073/pnas.1509070112 PMC4553775

[16] Ham J, Lim M, Kim D, Kim HY. Memory-like innate lymphoid cells in the pathogenesis of asthma. Front Immunol. 2022;13:1005517. 36466877 10.3389/fimmu.2022.1005517 PMC9712946

[17] Hsu AT, Gottschalk TA, Tsantikos E, Hibbs ML. The role of innate lymphoid cells in chronic respiratory diseases. Front Immunol. 2021;12:733324. 34630416 10.3389/fimmu.2021.733324 PMC8492945

[18] Kia L, Hirano I. Distinguishing GERD from eosinophilic esophagitis: concepts and controversies. Nat Rev Gastroenterol Hepatol. 2015;12:379–86. 25986303 10.1038/nrgastro.2015.75 PMC4948861

[19] Masuda MY, Pyon GC, Luo H, LeSuer WE, Putikova A, Dao A, et al. Epithelial overexpression of IL-33 induces eosinophilic esophagitis dependent on IL-13. J Allergy and Clin Immunol. 2024;153:1355–68. 10.1016/j.jaci.2024.01.017 PMC11070306 38310974

[20] Judd LM, Heine RG, Menheniott TR, Buzzelli J, O’Brien-Simpson N, Pavlic D, et al. Elevated IL-33 expression is associated with pediatric eosinophilic esophagitis, and exogenous IL-33 promotes eosinophilic esophagitis development in mice. Am J Physiol Gastrointest Liver Physiol. 2016;310:G13–25. 26514775 10.1152/ajpgi.00290.2015

[21] Travers J, Rochman M, Caldwell JM, Besse JA, Miracle CE, Rothenberg ME. IL-33 is induced in undifferentiated, nondividing esophageal epithelial cells in eosinophilic esophagitis. Sci Rep. 2017;7:17563. 29242581 10.1038/s41598-017-17541-5 PMC5730585

[22] Barrett NA, Shalek AK. Revisiting airway epithelial remodeling in type 2 immunity: beyond goblet cell metaplasia. J Allergy Clin Immunol. 2019;144:1158–60. 31600548 10.1016/j.jaci.2019.09.017

[23] Gieseck RL 3rd, Wilson MS, Wynn TA. Type 2 immunity in tissue repair and fibrosis. Nat Rev Immunol. 2018;18:62–76. 28853443 10.1038/nri.2017.90

[24] Nussbaum JC, Van Dyken SJ, von Moltke J, Cheng LE, Mohapatra A, Molofsky AB, et al. Type 2 innate lymphoid cells control eosinophil homeostasis. Nature. 2013;502:245–8. 24037376 10.1038/nature12526 PMC3795960

[25] Wee P, Wang Z. Epidermal growth factor receptor cell proliferation signaling pathways. Cancers (Basel). 2017;9:52. 28513565 10.3390/cancers9050052 PMC5447962

[26] Franke TF, Yang SI, Chan TO, Datta K, Kazlauskas A, Morrison DK, et al. The protein kinase encoded by the Akt proto-oncogene is a target of the PDGF-activated phosphatidylinositol 3-kinase. Cell. 1995;81:727–36. 7774014 10.1016/0092-8674(95)90534-0

[27] Gao J, Xia R, Chen J, Gao J, Luo X, Ke C, et al. Inhibition of esophageal-carcinoma cell proliferation by genistein via suppression of JAK1/2-STAT3 and AKT/MDM2/p53 signaling pathways. Aging (Albany NY). 2020;12:6240–59. 32276266 10.18632/aging.103019 PMC7185096

[28] Dowell J, Minna JD, Kirkpatrick P. Erlotinib hydrochloride. Nat Rev Drug Discov. 2005;4:13–4. 15690599 10.1038/nrd1612

[29] Barbera M, di Pietro M, Walker E, Brierley C, MacRae S, Simons BD, et al. The human squamous esophagus has widespread capacity for clonal expansion from cells at diverse stages of differentiation. Gut. 2015;64:11–9. 24572143 10.1136/gutjnl-2013-306171 PMC4283695

[30] Cheng E, Souza RF, Spechler SJ. Tissue remodeling in eosinophilic esophagitis. Am J Physiol Gastrointest Liver Physiol. 2012;303:G1175–87. 23019192 10.1152/ajpgi.00313.2012 PMC3532456

[31] Blanchard C, Mingler MK, Vicario M, Abonia JP, Wu YY, Lu TX, et al. IL-13 involvement in eosinophilic esophagitis: transcriptome analysis and reversibility with glucocorticoids. J Allergy Clin Immunol. 2007;120:1292–300. 18073124 10.1016/j.jaci.2007.10.024

[32] Zuo L, Fulkerson PC, Finkelman FD, Mingler M, Fischetti CA, Blanchard C, et al. IL-13 induces esophageal remodeling and gene expression by an eosinophil-independent, IL-13R alpha 2-inhibited pathway. J Immunol 2010;185:660–9. 20543112 10.4049/jimmunol.1000471 PMC3746758

[33] Huang Y, Guo L, Qiu J, Chen X, Hu-Li J, Siebenlist U, et al. IL-25-responsive, lineage-negative KLRG1(hi) cells are multipotential ‘inflammatory’ type 2 innate lymphoid cells. Nat Immunol. 2015;16:161–9. 25531830 10.1038/ni.3078 PMC4297567

[34] Huang Y, Paul WE. Inflammatory group 2 innate lymphoid cells. Int Immunol. 2016;28:23–8. 26232596 10.1093/intimm/dxv044 PMC4715228

[35] Cobb LM, Verneris MR. Therapeutic manipulation of innate lymphoid cells. JCI Insight. 2021;6:e146006. 33749662 10.1172/jci.insight.146006 PMC8026185

[36] Hosur V, Low BE, Shultz LD, Wiles MV. Genetic deletion of amphiregulin restores the normal skin phenotype in a mouse model of the human skin disease tylosis. Biol Open. 2017;6:1174–9. 28655741 10.1242/bio.026260 PMC5576083

[37] Byeon HK, Ku M, Yang J. Beyond EGFR inhibition: multilateral combat strategies to stop the progression of head and neck cancer. Exp Mol Med. 2019;51:1–14. 30700700 10.1038/s12276-018-0202-2 PMC6353966

[38] Maron SB, Xu J, Janjigian YY. Targeting EGFR in esophagogastric cancer. Front Oncol. 2020;10:553876. 33364187 10.3389/fonc.2020.553876 PMC7753114

[39] Morimoto Y, Hirahara K, Kiuchi M, Wada T, Ichikawa T, Kanno T, et al. Amphiregulin-producing pathogenic memory T helper 2 cells instruct eosinophils to secrete osteopontin and facilitate airway fibrosis. Immunity. 2018;49:134–50.e6. 29958800 10.1016/j.immuni.2018.04.023

[40] Mesnil C, Raulier S, Paulissen G, Xiao X, Birrell MA, Pirottin D, et al. Lung-resident eosinophils represent a distinct regulatory eosinophil subset. J Clin Invest. 2016;126:3279–95. 27548519 10.1172/JCI85664 PMC5004964

[41] Halim TY, Steer CA, Matha L, Gold MJ, Martinez-Gonzalez I, McNagny KM, et al. Group 2 innate lymphoid cells are critical for the initiation of adaptive T helper 2 cell-mediated allergic lung inflammation. Immunity. 2014;40:425–35. 24613091 10.1016/j.immuni.2014.01.011 PMC4210641

