# Supplementary material for: Esophageal ILC2s mediate abnormal epithelial remodeling in eosinophilic esophagitis via Areg-EGFR signaling
Source: Cell Mol Immunol. 2024 Dec 9;22(1):97–110. doi: 10.1038/s41423-024-01242-x (PMC11685411; doi:10.1038/s41423-024-01242-x)
Supplement: Supplementary file 1 — Supplementary figure and tables [file 41423_2024_1242_MOESM1_ESM.docx]

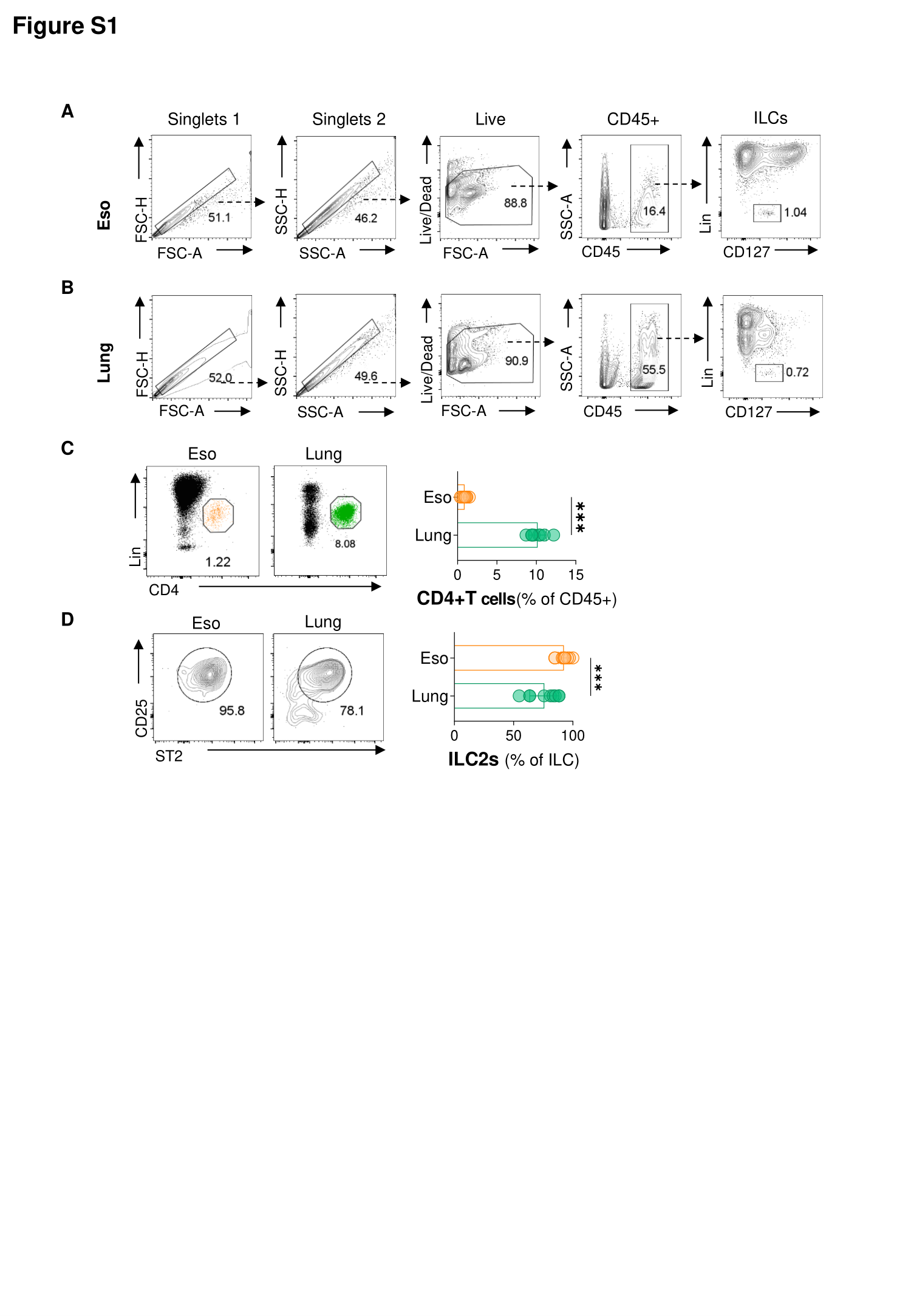


**Figure S1. Comparison of Innate Lymphoid Cells (ILCs) between Esophagus and Lung Immune Cells in Naïve Mice.**

(A-B) Representative gating strategy for identifying esophageal ILCs (A) and lung ILCs (B) by flow cytometry. (C) FACS plots and frequencies of CD4+ T cells in CD45+ total immune cells isolated from the esophagus and lung. (D) FACS plots and frequencies of CD25+ ST2+ ILC2s in Lin- CD127+ total ILCs in the esophagus and lung. Data are pooled from at least 2-3 independent experiments and are presented as the mean ± SEM. Statistical significance is denoted as *P ≤ 0.05, **P ≤ 0.01, ***P ≤ 0.001, and ****P ≤ 0.0001, while "ns" indicates not significant.

**
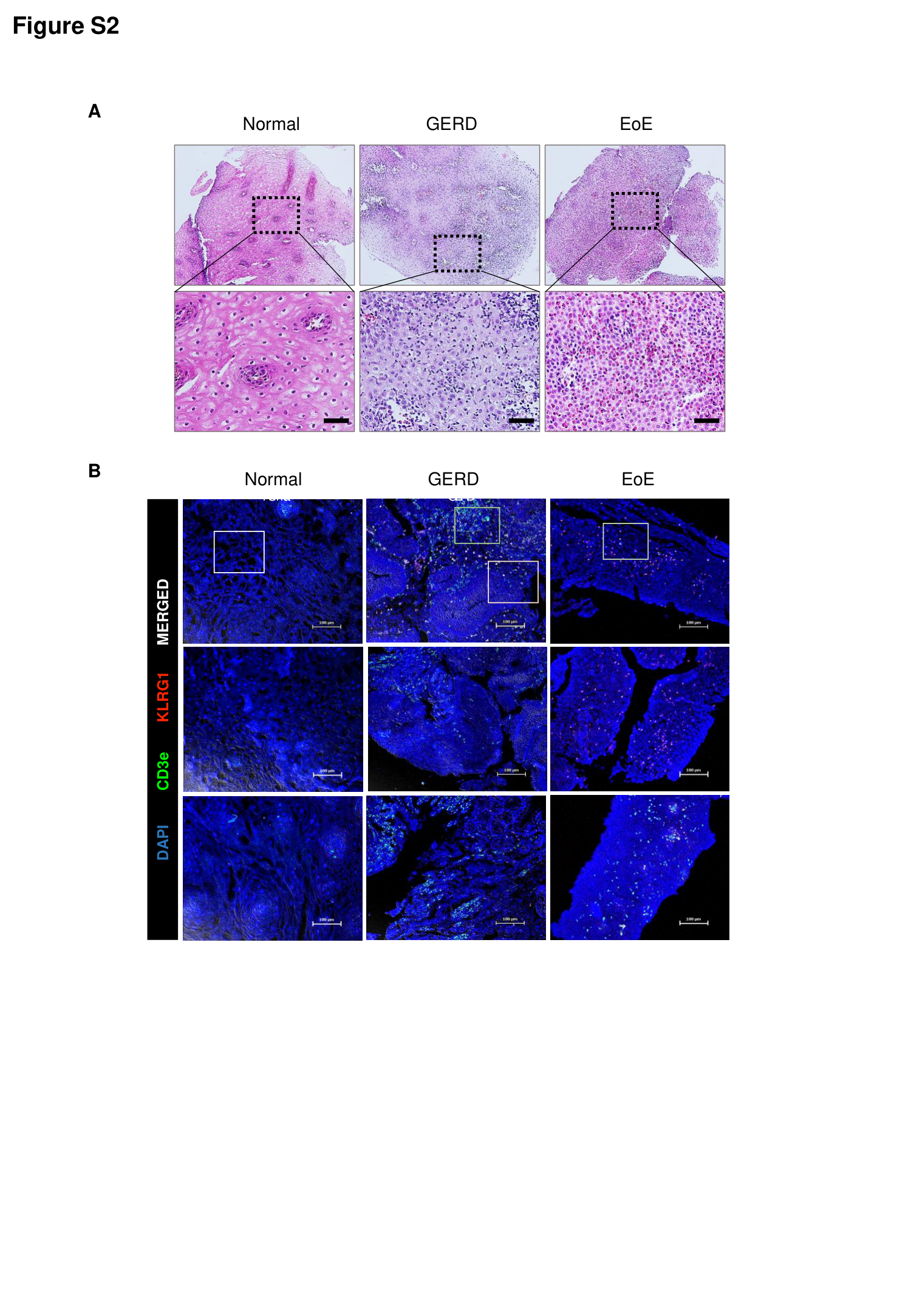
Figure S2. Identification of Esophageal ILC2s, Th2, and T cells using Immunofluorescence (IF) Images in Various Esophageal Diseases.**

(A) Representative hematoxylin and eosin images showing normal esophagus, gastroesophageal reflux disease (GERD), and eosinophilic esophagitis (EoE) cases. GERD exhibits increased lympho-histiocytic infiltration with basal cell hyperplasia, while EoE shows marked eosinophilic infiltration. Scale bar = 50 μm. (B) Uncropped IF images depicting KLRG1 (Red), CD3e (Green), and DAPI (Blue) staining in healthy control and various esophageal disease patient samples. Scale bars=100 μm.


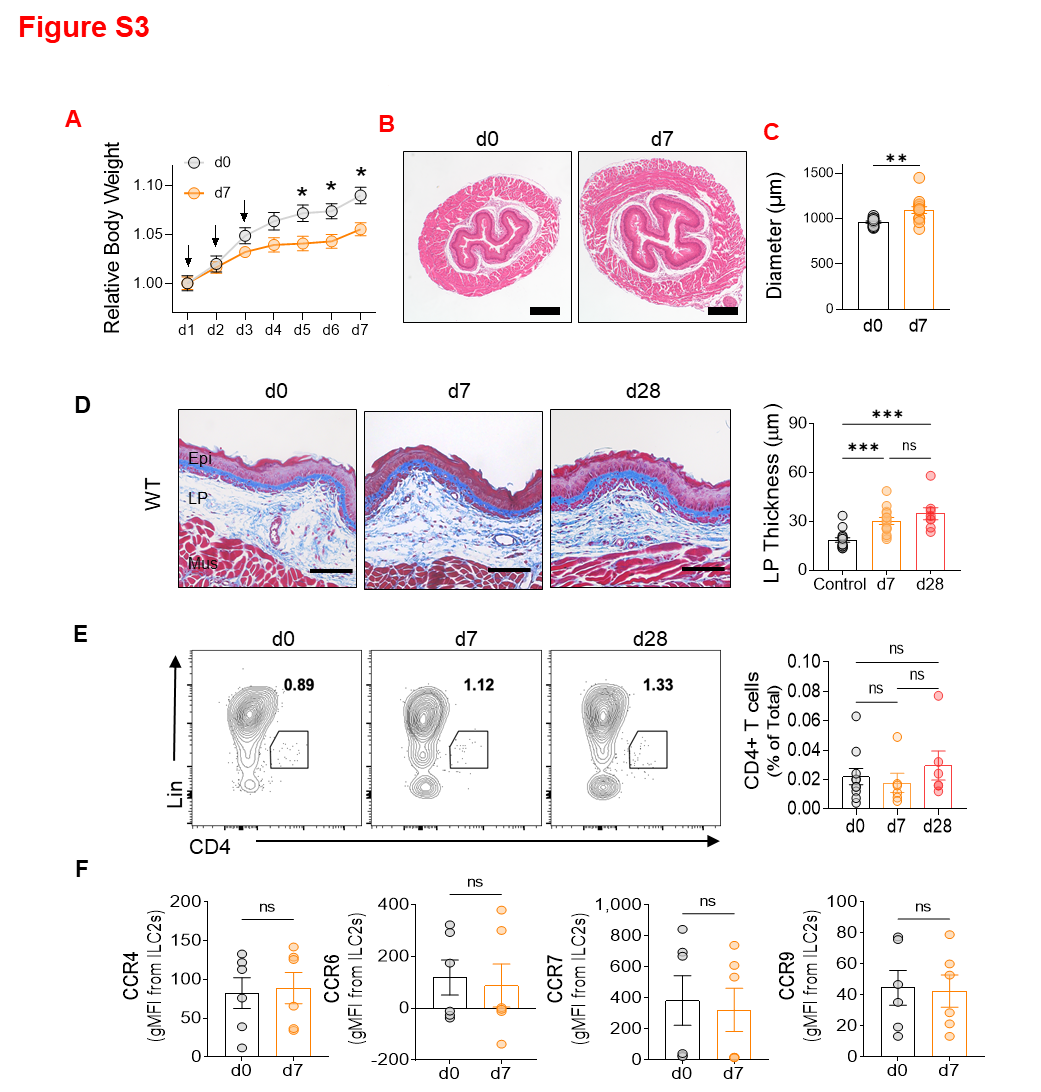


**Figure S3. Comparisons of the phenotypes of ILCs and CD4+ T cells in the Lamina Propria during EoE Development.**

(A) Changes in body weight in the IL-33-induced acute EoE model. (B) Representative cross-sections of the esophagus. Scale bars=200 µm. (C) Quantification of esophageal diameter. (D) Masson-trichrome-stained sections of the esophagus in EoE. Epithelium (Epi), Lamina Propria (LP), and Muscle (Mus). Scale bars =100 μm. (E) FACS plots and frequencies of CD4+ T cells resident in the esophagus during EoE. (F) Comparisons of chemokine receptors (CCR4, CCR6, CCR7, and CCR9) on esophageal CD25+ ST2+ ILC2s during acute EoE. Data are pooled from at least 2-3 independent experiments and presented as the mean ± SEM. Statistical significance is denoted as *P ≤ 0.05, **P ≤ 0.01, ***P ≤ 0.001, ****P ≤ 0.0001, and "ns" indicates not significant.


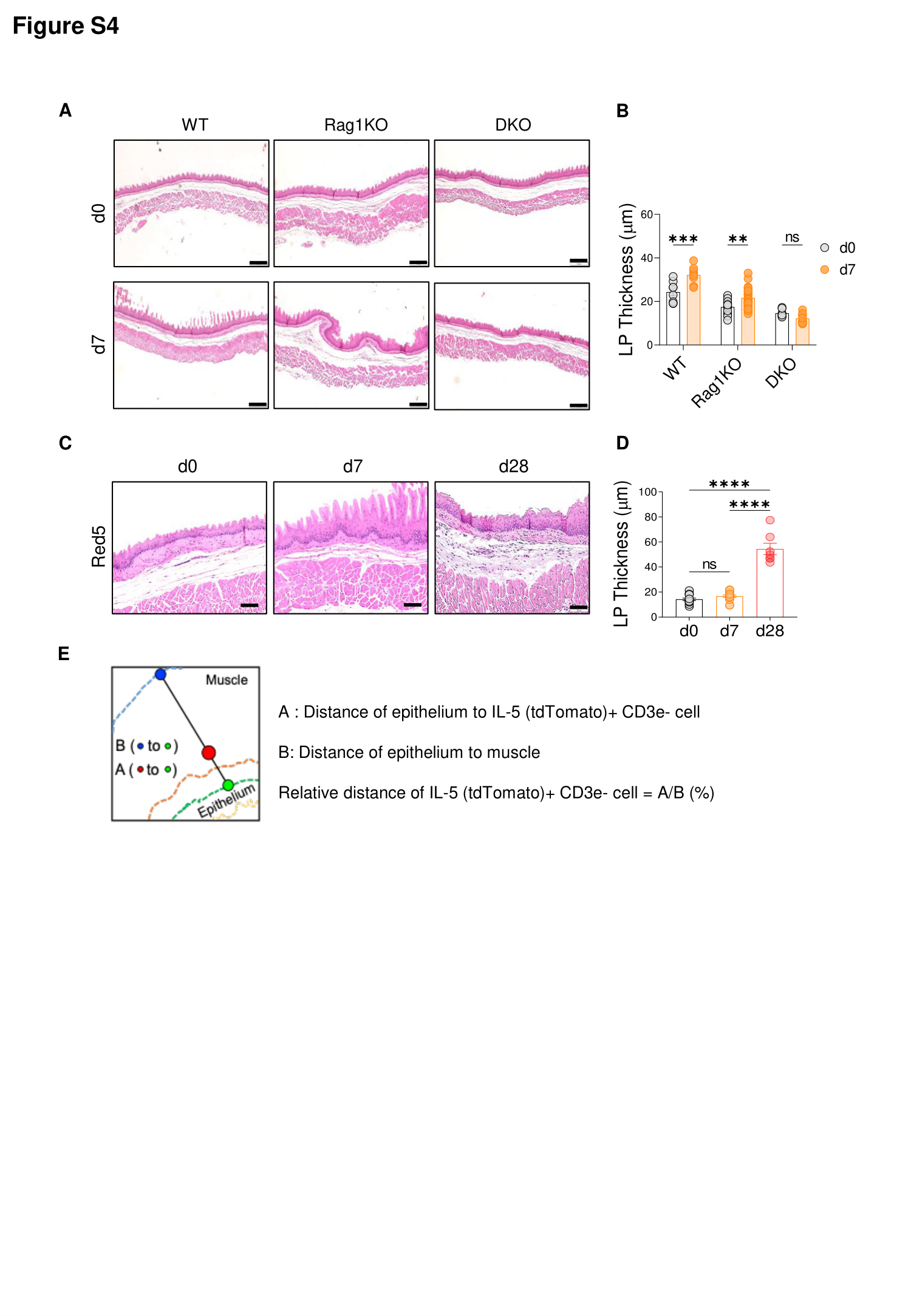


**Figure S4. Representative original images of H&E sections of EoE and esophageal lamina propria.**

(A) Original images of H&E sections showing control and acute EoE (wild-type, Rag1KO, and DKO mouse). Scale bars=200 μm. (B) Quantification of esophageal lamina propria thickness. (C) Original images of H&E sections of control, acute, and chronic EoE (Red5 mouse) Scale bars=100μm. (D) Quantification of esophageal lamina propria thickness. (E) Graphical representation of the measurement of CD3e- IL-5 (tdTomato)+ cells positioning at the esophageal epithelium during acute and chronic EoE. Blue dots represent the edge of the Esophageal muscle, red dots represent CD3e- IL-5+ ILC2s, and green dots represent the bottom of the epithelium. The blue dashed line indicates the outermost boundary of the muscle, while the yellow dashed line indicates the top of the epithelium. Localization of CD3e^−^IL-5^+^ ILC2s. A: The distance from the epithelium to IL-5 (tdTomato)+ CD3e− ILC2s. B: The distance from the epithelium to the muscle. The relative position of ILC2s from the epithelium is calculated as A/B (%). Data are pooled from at least 2-3 independent experiments and presented as the mean ± SEM. Statistical significance is denoted as *P ≤ 0.05, **P ≤ 0.01, ***P ≤ 0.001, ****P ≤ 0.0001, and "ns" indicates not significant.

**
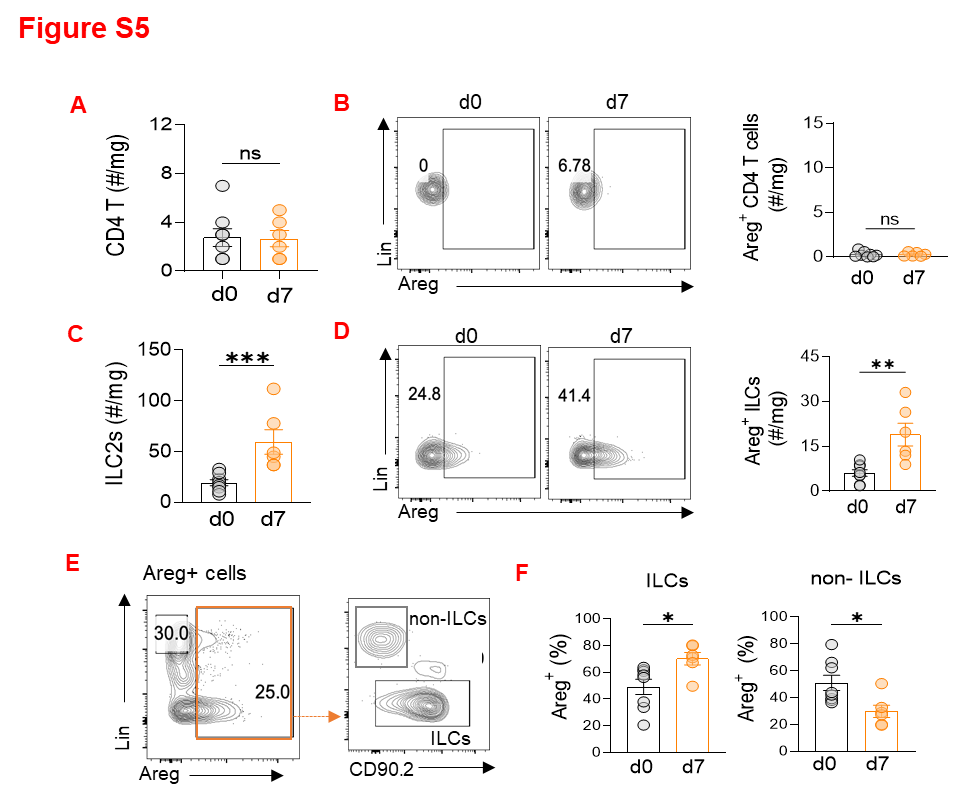
**

**Figure S5. Comparison of Areg production by ILC2s and T cells in IL-33 induced acute EoE mice model.**

(A) Absolute count of CD4^+^ T cells in the model (B) Representative flowcytometry plots and absolute count of Areg^+^ CD4^+^ T cells (C) Absolute count of ILC2s (D) Representative flowcytometry plots and absolute count of Areg^+^ ILC2s. (E) Representative flowcytometry plots Areg-producing cells (F) frequencies of Areg^+^ ILCs and non-ILCs. Data are shown as mean ± SEM. Statistical significance was determined using one-way ANOVA; *, P < 0.05; **, P < 0.01; ***, P < 0.001.

**
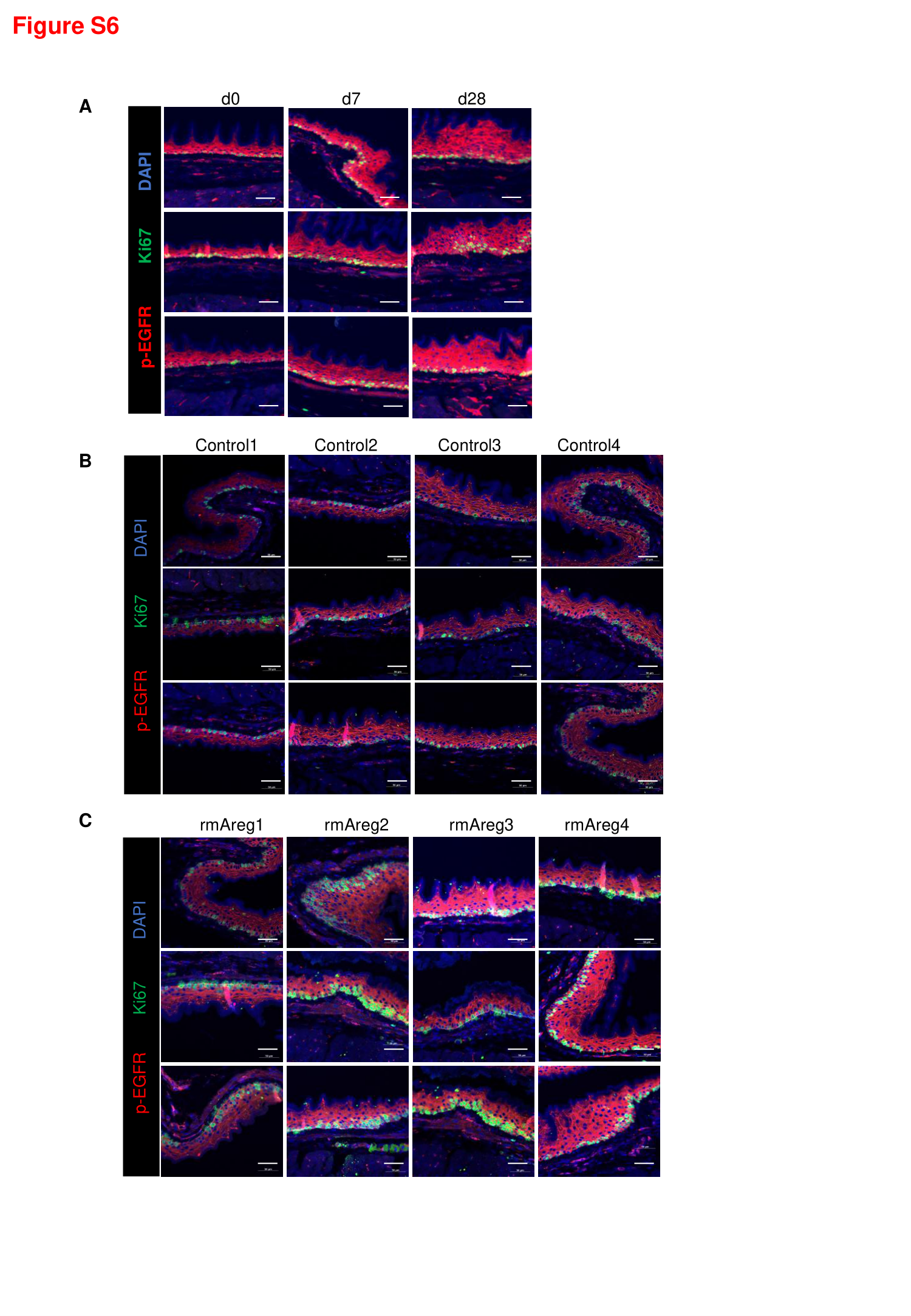
**

**Figure S6. Validation of basal cells hyperplasia and increased epithelium thickness under IL-33 induced EoE and rmAreg administration.**

(A) Original IF image analysis of the esophageal epithelium and basal cell hyperplasia in EoE (phospho-EGFR as Red, Ki67 as Green, DAPI as Blue). (B) Original IF image analysis of the esophageal epithelium and basal cell hyperplasia in control mice. (C) Original IF image analysis of the esophageal epithelium and basal cell hyperplasia in rmAreg-treated mice (phospho-EGFR as Red, Ki67 as Green, DAPI as Blue). All scale bars=50μm.

**
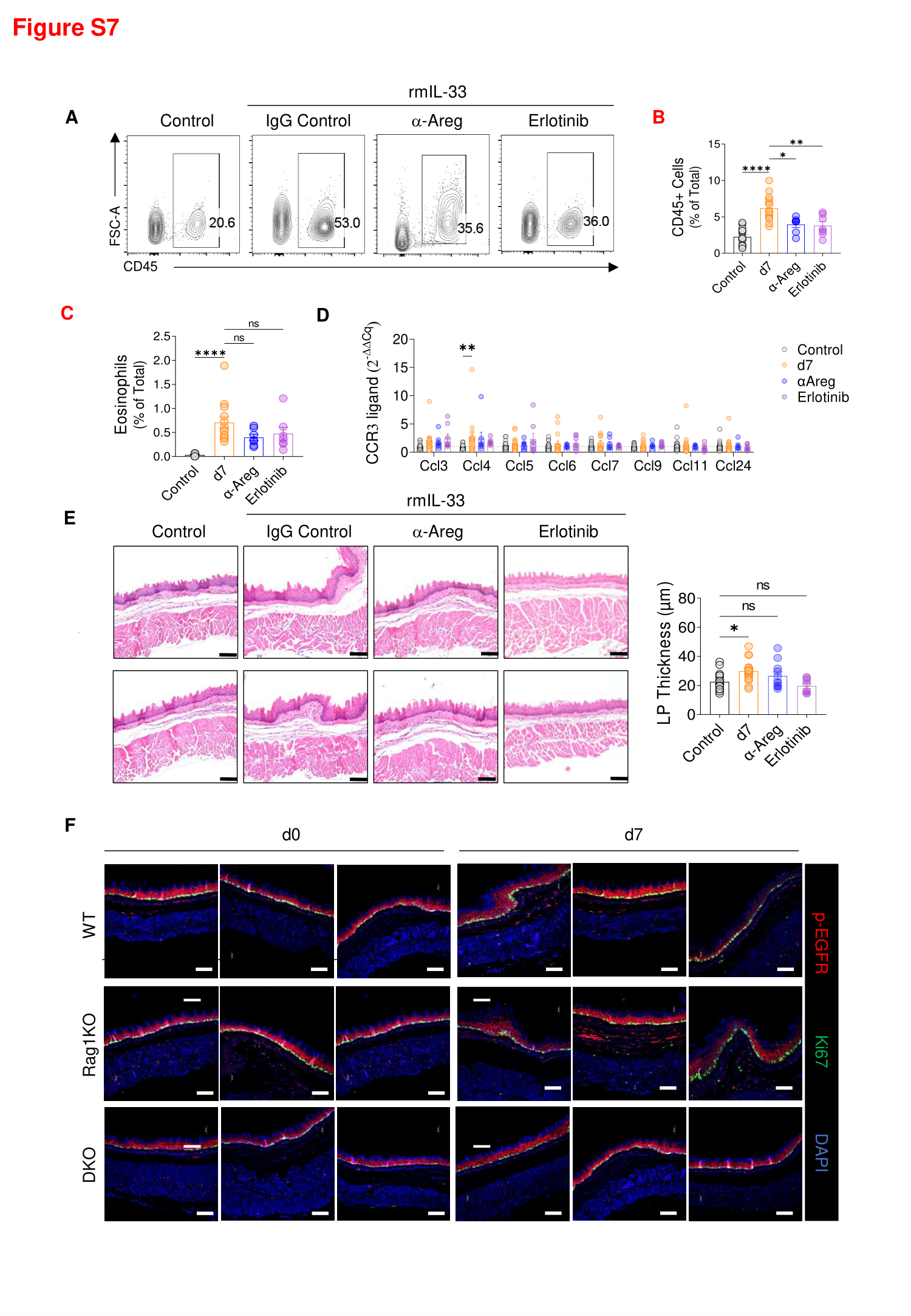
Figure S7. Inhibition of Areg-Egfr signaling and deficient of ILC2s reduced EoE phenotypes under EoE conditions.**



(A) Flow cytometry plots showing control, acute EoE, αAreg, and erlotinib-treated EoE mice esophageal CD45+ immune cells. (B) Frequencies of immune cells and (C) eosinophils in the esophagus following αAreg and erlotinib treatment. (D) Comparisons of CCR3-associated chemokines gene expression in whole mouse esophageal tissues under the inhibition of epidermal growth factor receptor (EGFR) signals during EoE. (E) Original images of hematoxylin and eosin (H&E) sections depicting acute EoE, αAreg, and erlotinib-treated EoE, with quantification of esophageal lamina propria thickness. Scale bars=200 μm. (F) Original IF image analysis of esophagus epithelium and basal cells hyperplasia in the WT, Rag1KO, and DKO mice with EoE (phospho-EGFR as Red, Ki67 as Green, DAPI as Blue). All scale bars=100 μm. Data are pooled from at least 2-3 independent experiments and presented as the mean ± SEM. Statistical significance is denoted as *P ≤ 0.05, **P ≤ 0.01, ***P ≤ 0.001, ****P ≤ 0.0001, and "ns" indicates not significant.

**
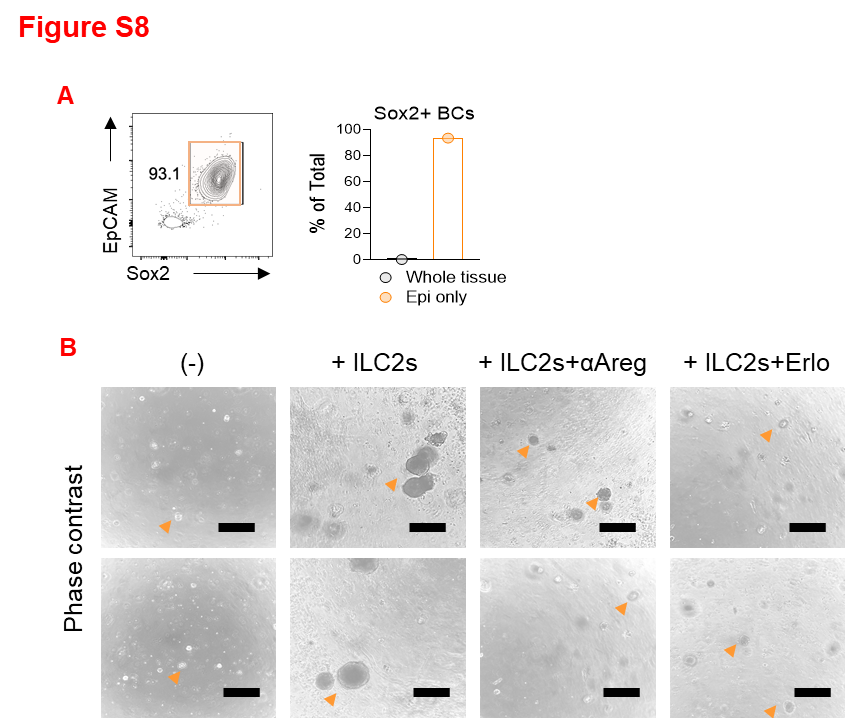
**

**Figure S8. Representative phase contrast images of the esophageal epithelial organoids**

(A) The purity of esophagus epithelial basal cells (BCs; CD31^−^CD45^−^EpCAM^+^Sox2^+^). (B) Representative phase contrast images of the esophageal epithelial organoids across different experimental groups. Orange arrows indicate organoid. Scale bars = 100µm.

**Supplemental Table 1.** Clinical characterization of Healthy control, GERD, and EoE Patients.

|  | **Control** | **EoE** | **GERD** |
| --- | --- | --- | --- |
| **Numbers of patients** | 8 (20%) | 22 (55%) | 10 (25%) |
| **Age (Year)** | 61.0±9.2 | 39.2±20.3 | 59.6±6.4 |
| **Sex (F/M)** | 1/8 | 6/22 | 3/10 |
| **Symptoms** |  |  |  |
| **Dysphagia, n (%)** | 3 (37.5%) | 8 (36.4%) | 1 (20%) |
| **Epigastric Pain, n (%)** | 3 (37.5%) | 11 (50%) | 2 (20%) |
| **Heart Burn, n (%)** | 2 (25%) | 3 (13.6%) | 4 (40%) |
| **Dyspepsia, n (%)** | 0 (0%) | 6 (27.3%) | 3 (30%) |
| **Patients’ history** |  |  |  |
| **Asthma, n (%)** | 0 (0%) | 2 (9.1%) | 1 (10%) |
| **Atopic Dermatitis, n (%)** | 0 (0%) | 1 (4.5%) | 0 (0%) |
| **Food Allergy, n (%)** | 0 (0%) | 1 (4%) | 0 (0%) |
| **Smoking History, n (%)** | 4 (50%) | 3 (13.6%) | 4 (40%) |
| **Alcohol History, n (%)** | 6 (75%) | 7 (31.8%) | 4 (40%) |
| **Previous PPI Use, n (%)** | 3 (37.5%) | 2 (9.1%) | 4 (40%) |

The data are presented as mean ± standard deviation of *n* (%)

**Supplemental Table 2.** Clonal information of flowcytometry antibody.

| Analyte detector | Reporter | Clone |
| --- | --- | --- |
| anti-CD45 | BV650 | 30-F11 |
| anti-CD19 | FITC | 1D3 |
| anti-CD49b | FITC | DX5 |
| anti-F4/80 | FITC | BM8 |
| anti-FcεR1a | FITC | MAR-1 |
| anti-CD127 | PE-Cy7 | A7R34 |
| anti-CD90.2 | PE-Cy7 | 30-H12 |
| anti-CD25 | BV785 | PC61 |
| anti-IFN-𝜸 | PerCP-Cy5.5 | XMG1.2 |
| anti-IL-17A | BV421 | TC11-18H10.1 |
| anti-IL-5 | APC | TRFK5 |
| anti-ICOS | BV785 | C398.4A |
| anti-KLRG1 | PerPC-Cy5.5 | 2F1 |
| anti-SCA1 | BV785 | D7 |
| anti-Streptavidin | PerCP-Cy5.5 | 405214 |
| anti-CCR3 | PE-Cy7 | J073E5 |
| anti-CCR9 | PE | 9B1 |
| anti-CCR7 | BV785 | 4B12 |
| anti-CCR4 | BV421 | 2G12 |
| anti-CCR6 | APC | 29-2L17 |
| anti-SiglecF | APC | S17007L |
| anti-Gata3 | BV421 | L50-823 |
| anti-Tbet | PE | 4B10 |
| anti-Ror𝜸t | APC | B2D |
| anti-Areg | biotin | Polyclonal |
| anti-IL-13 | PE | ebio13A |
| anti-ST2 | biotin | RMST2-33 |
| anti-CD3e | FITC | 145-2C11 |
| anti-CD11b | FITC | M1/70 |
| anti-CD11c | FITC | HL3 |
| anti-CD4 | BV785 | RM4-5 |
| anti-CD31 | BV421 | MEC 13.3 |
| anti-EpCAM | PE-Cy7 | G8.8 |
| anti-Sox2 | PerCP Cy5.5 | O30-678 |
